# Dual-task interference in the drop jump: Frequentist, Bayesian, and waveform analysis of vertical ground reaction force

**DOI:** 10.1371/journal.pone.0362399

**Published:** 2026-11-11

**Authors:** Huseyin Celik, Pinar Arpinar-Avsar

**Affiliations:** Department of Biomechanics and Motor Control, Faculty of Sport Sciences, Hacettepe University, Ankara, Türkiye; Ordu University, TÜRKIYE

## Abstract

To ensure standardization, reliability, and maximal performance, physical performance tests are usually performed with little to no cognitive load. However, during training and competition, athletes usually perform physical actions while under the demands of a concurrent cognitive task (CT). This study examined the effect of a concurrent CT on the force-time metrics of the drop jump (DJ) and their reliability under single-task (DJ without a CT) and dual-task conditions (DJ with a CT based on simple arithmetic calculations). Thirty-five healthy young participants performed single-task and dual-task DJ trials in a randomized order. Nineteen force-time metrics covering the braking, propulsion, and landing phases of the DJ were compared between conditions using paired t-tests with Holm adjustment and Hedges’ $g_{z}$, Bayesian paired t-tests, and statistical parametric mapping (SPM) of vertical ground reaction force (VGRF) waveforms. For three-trial mean scores, 17 of the 19 metrics had within-session intraclass correlation coefficients (ICCs) of 0.812–0.930 across both conditions. Under the dual-task condition, jump height (p < 0.001, $g_{z}$=1.40, Bayes factor *BF*_10_ = 1.76×10^7^) and reactive strength index (p < 0.001, $g_{z}$=1.04, *BF*_10_ = 45,943) were lower, while ground contact time was longer (p = 0.025, $g_{z}$=−0.57, *BF*_10_ = 20.57). SPM showed that VGRF was significantly lower under the dual-task condition during 29.2–34.3% (within braking) and 52.0–79.1% (within propulsion) of ground contact. In the landing phase, VGRF was significantly lower under the dual-task condition during 1.3–6.3% (initial force rise) and significantly higher during 15.5–19.6% (attenuation of the impact force). The spring-like correlation (p = 0.462, $g_{z}$=0.20, *BF*_01_ = 2.79) and the vertical time to stabilization (p = 0.462, $g_{z}$=0.15, *BF*_01_ = 3.74) showed no significant difference between conditions, with weak and moderate evidence for the absence of an effect. These findings suggest that a concurrent CT reduces force production within both the braking and propulsion phases of the DJ and alters the VGRF waveform during the subsequent landing phase in healthy young participants.

## Introduction

The drop jump (DJ) is a well-established test of stretch-shortening cycle (SSC) function and is widely used in athlete monitoring, strength and conditioning, and readiness-to-train assessment [1]. The DJ imposes rapid eccentric loading upon landing, requiring the neuromusculoskeletal system to absorb and rapidly reuse energy within a brief ground contact window through a fast SSC [2]. Neuromuscular control of the DJ combines anticipatory, largely pre-programmed (feedforward) muscle activation before touchdown with stretch-reflex and central contributions later during ground contact [3]. Force plates enable comprehensive analysis of the biomechanical response across the braking and propulsion phases of ground contact and the subsequent landing phase through phase-specific force-time metrics [4] and continuous force-time waveform analysis [5]. Together, these analyses can characterize lower-limb biomechanics at a level of detail that scalar metrics such as jump height (JH) cannot capture [4,6].

To ensure standardization, reliability, and maximal performance, physical performance tests are usually conducted with little to no cognitive load [7,8]. However, during training and competition, athletes usually perform motor tasks concurrently with cognitive tasks (CTs). For instance, they may scan the field to make decisions while jumping, landing, or changing direction [9]. Limited-capacity theory proposes that attention can be allocated between concurrent activities, although the total capacity available at any time is limited [10]. Multiple-resource theory further proposes that interference depends on both the demands of each task and the processing resources they share [11]. The magnitude of cognitive-motor interference may therefore depend on task demands, resource overlap, and how attention is allocated between tasks. Under dual-task conditions, motor performance, cognitive performance, or both may deteriorate [12]. The effects of a concurrent CT on motor performance can be examined by comparing a motor task performed alone in a single-task condition with the same motor task performed concurrently with a CT in a dual-task condition [13].

Anticipatory neuromuscular control relies on motor preparation before movement onset, and this preparation draws on central processing resources. For instance, in a reaction-time experiment using this design, the state of motor preparation was lower even for a simple, single-joint wrist-extension response when participants counted backward concurrently than when they performed the wrist response alone [14]. In a more complex, whole-body motor task such as the drop vertical jump, single- and dual-task comparisons have also shown that a concurrent CT alters jump performance [8]. Force during early ground contact is influenced by preparatory muscle activation and the geometrical configuration of the lower limbs at touchdown [3]. However, VGRF alone cannot distinguish the contributions of anticipatory, reflex-mediated, and voluntary control. Phase-specific and continuous waveform analyses have the potential to identify single- and dual-task differences during different phases of the DJ and across the force-time waveform that cannot be inferred from an overall outcome such as JH. However, comprehensive force-time analysis of the DJ under cognitive-motor dual-task conditions remains limited, with previous studies focusing mostly on overall performance outcomes or selected kinetic and kinematic variables rather than combining phase-specific metrics with continuous waveform analysis [9].

Cognitive demands have been added to DJs and jump-landing tasks in several ways. For instance, while continuous arithmetic CTs divide attention between the motor and cognitive tasks, visual or auditory cues can add decision-making demands or reduce the time available for motor planning [8,15–17]. The added cognitive demands examined in these studies have significantly altered jump performance and landing mechanics [8,15,16,18]. Much of the landing-mechanics research has been framed around biomechanical factors associated with anterior cruciate ligament (ACL) injury and return-to-sport screening, including recent time-series analyses of the first 100 ms after contact in athletes following ACL reconstruction [17,19]. However, research examining landing mechanics under a concurrent CT in healthy young adults remains limited outside the context of injury-related assessment [9].

Simple arithmetic calculations are commonly used as a CT in cognitive-motor dual-task experiments [e.g., 15]. For instance, Shumski et al. [8] used serial subtraction by sixes or sevens during bilateral drop vertical jumps from a 30 cm box and reported significantly lower JH (mean difference = 1.84 cm) and normalized hip power (mean difference = 0.29 W·BW^–1^·HT^–1^; BW, body weight; HT, height) under the dual-task condition, with no significant interaction between sex and condition among recreationally active young adults. Additionally, Dai et al. [15] examined healthy young recreational athletes who counted backward by ones or sevens during a jump-landing task and reported that JH was significantly lower and that peak posterior ground reaction force and peak vertical ground reaction force (VGRF) were significantly higher during the first 100 ms of landing. In another study, Imai et al. [18] used two-digit addition during a DJ performed by young female collegiate athletes and reported that hip and knee flexion at initial contact were significantly lower, while peak VGRF and several lower-extremity joint moments during the first 40 ms after contact were significantly higher. Using a different approach to impose cognitive demands, Holmes et al. [16] reported significantly longer ground contact time (GCT) in healthy young adults when audio and visual cues reduced the time available for motor planning during a 30 cm DJ. Previous studies have focused mostly on overall performance metrics, selected kinetic and kinematic variables, predefined early intervals after contact, or landing-focused time series. Research combining phase-specific force-time metrics with continuous VGRF waveform analysis across braking, propulsion, and the subsequent landing remains limited.

For long-term athlete monitoring, testing conditions should be standardized between sessions so that changes in performance can be interpreted and used to make informed decisions [7]. Standardization should include the measurement equipment used, testing environment, participant preparation, test protocol, and cognitive demands. If cognitive demands are not standardized, for instance, DJ test outcomes obtained under divided attention may reflect both the athlete’s physical performance and the attentional condition under which the test was performed. To assess the reproducibility of a test, reliability analyses quantify relative and absolute reliability using repeated measurements obtained under the same conditions [20]. The intraclass correlation coefficient (ICC) quantifies relative reliability, while the standard error of measurement (SEM) and coefficient of variation (CV) quantify absolute reliability. In particular, the SEM estimates measurement error in the original unit of the metric. Under conventional single-task testing, 16 force-time metrics from bilateral DJs, including vertical time to stabilization (VTTS), had intraday absolute-agreement ICCs of 0.78–0.89 and interday ICCs of 0.72–0.90 [21]. However, the intraday reliability of DJ force-time metrics under a concurrent CT remains understudied, and estimating it in both conditions would allow single- and dual-task differences to be interpreted in relation to within-session measurement error.

Discrete force-time metrics of the DJ, such as reactive strength index (RSI), cannot identify where single- and dual-task conditions differ across a normalized force-time waveform. Statistical parametric mapping (SPM) enables field-wide statistical inference across a continuous one-dimensional waveform while controlling the family-wise error rate [5,22]. Pedley et al. [23] applied SPM to DJ VGRF waveforms without a concurrent CT. Using SPM in dual-task landing research, Nguyen et al. [24] reported significantly greater hip adduction in the visual and auditory CT conditions during 88–100% of the landing period and significantly greater hip internal rotation in the visual CT condition during 68–92% compared with the single-task condition. However, application of SPM to DJ VGRF waveforms under a concurrent CT remains limited. Applying SPM across the braking, propulsion, and subsequent landing phases has the potential to identify significant clusters indicating differences between single- and dual-task VGRF waveforms.

Frequentist and Bayesian analyses can provide complementary information about single- and dual-task differences [25,26]. For instance, a non-significant p value indicates that the null hypothesis was not rejected, although it does not distinguish evidence for the absence of an effect from inconclusive evidence [26]. To address this limitation, the Bayes factor (BF) is used to quantify the relative evidence that the data provide for one hypothesis over another. A BF can therefore distinguish evidence for an effect, evidence for the absence of an effect, and inconclusive evidence [26]. This distinction is relevant to dual-task landing research because a recent systematic review and meta-analysis reported small and inconsistent biomechanical effects [27]. For one-dimensional biomechanical data, Bayesian SPM extends this approach by showing how strongly the data support a condition difference throughout the waveform [25]. Combining the two approaches therefore has the potential to identify significant clusters using frequentist SPM and evaluate the strength of evidence for differences across the waveform using Bayesian SPM.

Therefore, the purpose of this study was to investigate the effects of a concurrent CT on DJ force-time metrics and waveforms and to establish the intraday reliability of the metrics under single- and dual-task conditions. To this end, we compared 19 VGRF-derived force-time metrics between conditions using frequentist paired *t*-tests with Hedges’ $g_{z}$ and Bayesian paired *t*-tests. Additionally, *t*ime-normalized VGRF waveforms across the braking, propulsion, and subsequent landing phases were compared using frequentist and Bayesian SPM. For the reliability analysis, the ICC, SEM, and CV were estimated separately for each condition. Our first hypothesis was that motor performance during the DJ would deteriorate under the dual-task condition. We also expected the VGRF waveforms to differ between conditions during the braking, propulsion, and subsequent landing phases. Our second hypothesis was that the force-time metrics would provide reliable measurements from trial to trial under both single- and dual-task conditions.

## Materials and methods

### Participants

Thirty-five coaching education students with competitive athletic backgrounds (18 men and 17 women) participated in the experiments (age: 19.0 ± 2.5 years; height: 177.1 ± 6.9 cm; body mass: 68.1 ± 10.0 kg). Sports represented included volleyball (n = 13), soccer (n = 7), basketball (n = 4), handball (n = 2), swimming (n = 2), and other or unspecified sports (n = 7). Participants were eligible if they were aged 18–35 years, trained at a recreational or university team level, had at least two years of regular training experience with a minimum of three hours of exercise or sport per week, had prior experience in plyometric and strength training, and reported no health problems that would prevent maximal-effort jumping. Training status was consistent with Tier 1 (recreationally active) to Tier 2 (trained/developmental) [28], and the sample could therefore be described as healthy young recreational athletes.

Using G*Power [v. 3.1; 29], we performed an a priori statistical power analysis to determine the sample size required for a two-tailed paired-samples *t*-test comparing two dependent means. The calculation assumed a standardized paired effect size of $d_{z}$ = 0.50, consistent with the sample-size calculation of Dai et al. [15]. A minimum of 34 participants was required for 80% power at α = 0.05 for a single paired comparison. We recruited 35 volunteers to participate in the experiments.

Written informed consent was obtained from all participants before data collection. Participants were recruited and data were collected between October 1 and 31, 2024. The study procedures were approved by the Hacettepe University Health Sciences Research Ethics Board (research number SBA 23/274; decision 2023/07–44, 21 November 2023) and were conducted in accordance with the Declaration of Helsinki.

### Protocol

Participants performed the DJ in the akimbo position on a force plate (Kistler 9260AA6, Switzerland) at a 1000 Hz sampling rate, with a 30 cm drop height to balance external load and injury risk. The warm-up procedure consisted of five minutes of jogging followed by dynamic stretching of each major muscle group of the lower limbs. After the warm-up, participants were given sufficient time to become familiar with the test equipment and procedures and to practice DJs under single- and dual-task conditions. All participants were able to perform both conditions correctly before the recorded trials.

Each participant completed six trials in a single session, three in a single-task condition (DJ only) and three in a dual-task condition (DJ performed concurrently with a serial subtraction task). The order of the six trials was randomized for each participant using the random.org sequence generator (https://www.random.org/sequences/). Consecutive trials of the same condition were permitted. Approximately one minute of rest was provided between trials. In both conditions, participants were instructed to step forward off the box without stepping down or jumping up, and to land on both feet. Participants were verbally instructed to minimize GCT and jump as high as possible, with the force plate conceptualized as a hot plate [30]. After landing, participants were instructed to hold a stable position until the command to step off.

Throughout each trial, the hands were kept on the hips in the akimbo position, the most common arm position in jump testing [31]. This position limits the contribution of arm swing to jump performance. Restricting arm motion during landing also follows recommendations for improving the reliability of time-to-stabilization measurements [30].

Force-time data were acquired with the Kistler MARS interface and exported for subsequent analysis. Data acquisition was terminated automatically when the software detected the end of the jump sequence. The automatic stopping mode was selected to reflect the practical time constraints of team testing. Testing and data analysis followed the procedures of Celik et al. [21] and Pedley et al. [4].

In the dual-task condition, the researcher stated a randomly chosen number between 60 and 99 immediately before the jump. Participants counted backward aloud in steps of three from that number and continued subtracting from the moment they initiated the movement until the command to step off the platform. The task was adapted from Shumski et al. [8], who used subtraction by sixes or sevens from a number between 90 and 150. No instruction was given to prioritize either task. Trials were repeated if the participant stopped subtracting or made an error before the command to step off. A trial was also repeated if the participant left the platform before that command. The overall trial repetition frequency was below 5%, including restarts due to technical recording issues.

### Data processing

The VGRF signal was filtered using a second-order low-pass zero-lag Butterworth filter with a cutoff frequency of 100 Hz. Forward and reverse filtering removed phase lag. However, filtering can alter signal amplitudes and therefore the instants at which a force threshold is crossed. The cutoff follows previous DJ work that low-pass filtered VGRF at 100 Hz [32] and dual-task landing work that filtered ground reaction force at the same frequency [15]. All metrics were computed from the filtered signal.

To document the influence of this choice, the analysis was repeated on the unfiltered signal and at cutoff frequencies of 30 and 50 Hz. Filtering at 100 Hz reproduced the unfiltered impact peak and spring-like behavior classifications in all trials. Lower cutoff frequencies altered the classification (seven of the 13 impact peaks detected in the unfiltered data were attenuated at 30 Hz and four at 50 Hz).

Body mass was estimated by averaging the mean unfiltered VGRF over the final 100 ms of each of the six trials and dividing by gravitational acceleration (9.80665 m/s^2^). BW was calculated as body mass multiplied by gravitational acceleration.

The VGRF signal was then used to identify four key time points following the method described by Celik et al. [21]. The instant of drop landing (t2) was defined as the first sample at which VGRF exceeded 15 N [4]. The takeoff instant (t4) was the first sample at which VGRF dropped below 15 N after landing. The braking-to-propulsion transition (t3), which divides ground contact into the braking and propulsion phases, was identified as the last sample before the vertical velocity of the total body center of mass (BCOM) became non-negative, computed from the numerical integration of the net vertical force using the impulse-momentum theorem. The participant was assumed to be motionless at the end of the record. Velocity at t2 was therefore calculated as the negative of the net impulse from t2 to the end divided by body mass, and subsequent velocity was obtained by forward integration. Downward velocity and displacement were represented by negative values, while displacement metrics were reported as magnitudes. The rebound landing instant (t5) was the first sample exceeding 15 N in a search beginning 100 ms after takeoff, the delay excluding force transients immediately after the foot leaves the plate. The record was bounded by its first sample (t1), at which acquisition began, and its last sample (t6; Fig 1).

**Fig 1:**
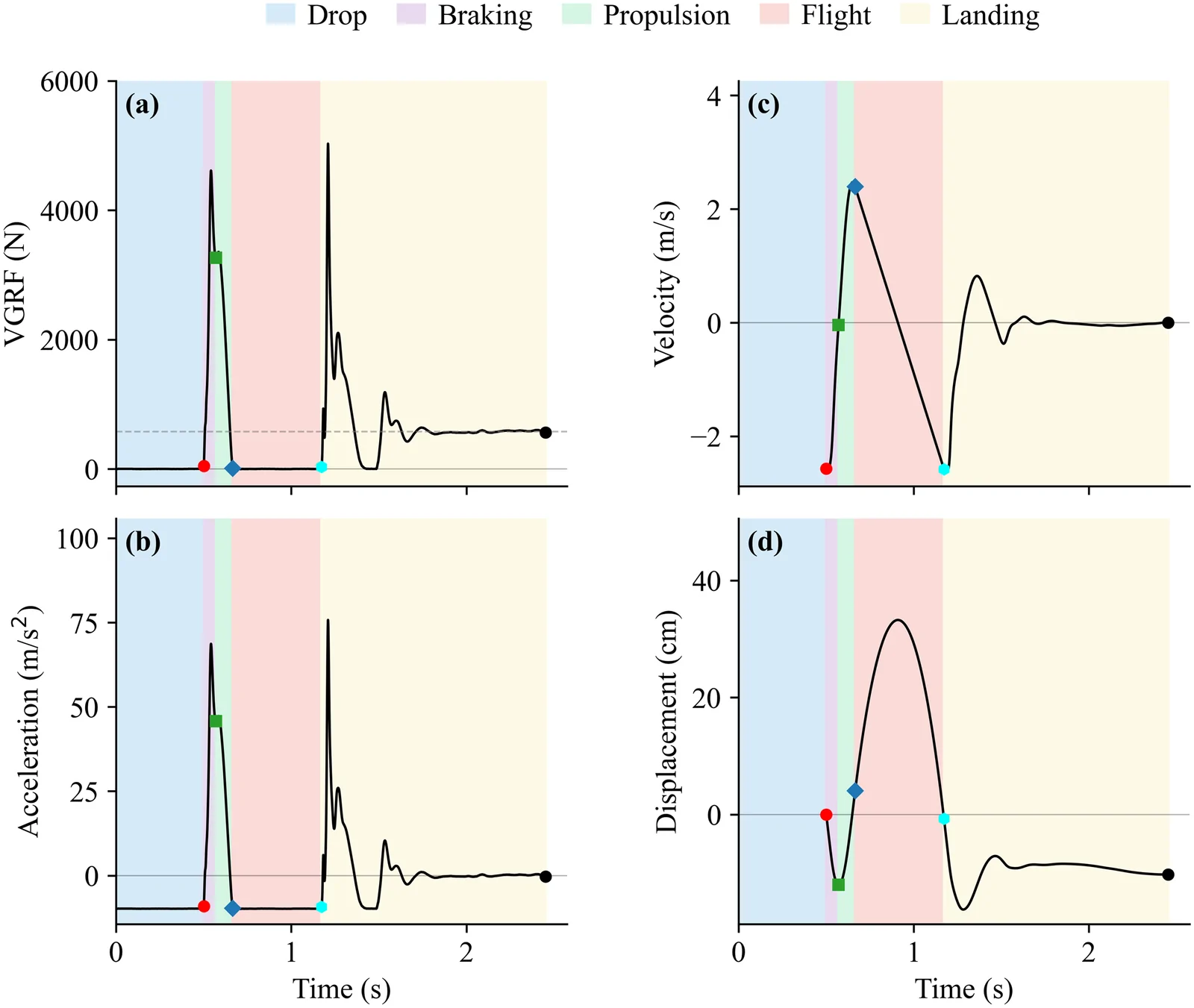
Example force-time and kinematic characteristics of a single-task drop jump. Panels show vertical ground reaction force (VGRF, a), body center of mass acceleration (b), velocity (c), and displacement (d). Shaded bands identify the drop, braking, propulsion, flight, and landing phases. Drop landing (t2) is marked by a red circle, the braking-to-propulsion transition (t3) by a green square, takeoff (t4) by a blue diamond, rebound landing (t5) by a cyan hexagon, and the end of recording (t6) by a black circle. Velocity and displacement were calculated from t2 onward, with upward defined as positive and displacement set to zero at t2. The dashed horizontal line in (a) indicates body weight.

After identifying these time points, 19 force-time metrics were estimated from the VGRF curves. Four metrics were selected for graphical presentation. These were JH, GCT, and RSI, which Bishop et al. [7] advise monitoring for the DJ, and peak force (PF) at landing, because prior cognitive-load landing studies reported changes in peak VGRF [15,33].

- **Jump height (JH, cm):** Vertical rise of the BCOM from takeoff to the flight apex, estimated as $g\times {flight\;time}^{2}/8$, assuming equal BCOM heights at takeoff and rebound landing. Here, *g* is gravitational acceleration.
- **Ground contact time (GCT, ms):** Total time on the ground from t2 to t4. GCT distinguishes fast SSC function (100–250 ms) from slow SSC function (>250 ms).
- **Reactive strength index (RSI, m/s):** Ratio of JH to GCT, which quantifies the ability to produce force rapidly during the SSC.
- **Average braking power (Avg Pow Braking, W/kg):** Mean mechanical power during the braking phase, calculated as the work done against BCOM displacement divided by braking phase duration, normalized to body mass.
- **Average propulsion power (Avg Pow Propulsion, W/kg):** Mean mechanical power during the propulsion phase, calculated as propulsion work divided by phase duration and normalized to body mass.
- **Peak BCOM displacement (Delta COM, cm):** Maximum downward displacement of the BCOM during ground contact, calculated from double integration of the net vertical acceleration.
- **Peak braking force (PF Braking, BW):** Maximum VGRF during the braking phase, normalized to BW.
- **Peak propulsion force (PF Propulsion, BW):** Maximum VGRF during the propulsion phase.
- **Landing-to-takeoff timing difference (LTTD, %):** Time interval between the peaks of PF Braking and PF Propulsion, expressed as a percentage of GCT. Smaller values indicate a more compact force profile characteristic of effective SSC function.
- **Normalized vertical stiffness (Kvert, N/m/kg):** Ratio of peak VGRF to BCOM displacement at the instant of peak VGRF, divided by body mass.
- **Braking phase time (BPT, ms):** Duration of the braking phase (t2 to t3).
- **Propulsion phase time (PPT, ms):** Duration of the propulsion phase (t3 to t4).
- **Braking impulse (Braking Imp, BW·s):** Area under the VGRF–time curve during the braking phase.
- **Propulsion impulse (Propulsion Imp, BW·s):** Area under the VGRF–time curve during the propulsion phase.
- **Spring-like correlation (SLC):** Pearson correlation coefficient between VGRF and vertical BCOM displacement during ground contact. Spring-like behavior was classified as good (SLC <−0.80 without an impact peak), moderate (SLC <−0.80 with an impact peak or SLC ≥ −0.80 without an impact peak), or poor (SLC ≥ −0.80 with an impact peak) [4,32]. Under the impact-peak definition used in the main analysis, the combination of SLC ≥ −0.80 without an impact peak was not observed in any of the 210 trials. An impact peak was identified when the global contact-phase VGRF maximum occurred within the first 20% of ground contact. Absolute SLC values were used in the statistical analyses, with larger values indicating greater spring-like behavior. The sensitivity of these classifications to the impact-peak definition is described in S1 Appendix.
- **Peak landing force (PF Landing, BW):** Maximum VGRF from rebound landing (t5) to the end of the recorded trial (t6).
- **Landing loading rate (LR Landing, BW/s):** Maximum rate of VGRF increase between t5 and the instant of PF Landing. Rates were calculated as the change in VGRF between the endpoints of a moving window spanning 5% of that interval, divided by window duration.
- **Landing impulse (Landing Imp, BW·s):** Area under the VGRF–time curve from t5 to VTTS.
- **Vertical time to stabilization (VTTS, s):** Time required to regain a stable standing posture after rebound landing (t5), estimated from VGRF [34]. VTTS was calculated using a fixed-window adaptation of the sequential estimation method of Colby et al. [35]. A 3 s post-touchdown reference window was used, and each recording was extended to 3 s using a constant equal to its final 100 ms mean VGRF. The implementation and its assumptions are described in S2 Appendix.

### Statistical analysis

#### Reliability.

Within-session (intraday) reliability was assessed separately for each condition using a two-way random-effects ANOVA on the three-trial data matrix (participants × trials) [21]. Relative reliability was quantified by the ICC, in the ICC(2,k) absolute agreement form with k = 3 trials, with 95% confidence intervals derived from the F distribution [36].

Absolute reliability was quantified by the SEM and the CV. The SEM was taken as the square root of the residual mean square, which estimates residual error for a single trial and therefore excludes the systematic between-trial component that the absolute-agreement ICC includes. The CV was computed as the mean of the individual participant CV values [37]. Individual CVs were calculated from the second and third trials of each condition. All three trials were used for the ICC and SEM. The sampling distribution of the mean participant CV was estimated by bootstrap resampling with replacement [38]. Participants were resampled 20,000 times. The 2.5th and 97.5th percentiles of the bootstrap means defined the 95% confidence interval. These intervals quantify uncertainty in the mean of the two-trial participant CVs.

Relative reliability was interpreted using the ICC estimates and their 95% confidence intervals. Absolute measurement error was evaluated using SEM and CV, with consideration of the magnitude of the observed condition differences.

#### Frequentist analysis.

Condition differences were tested using two-tailed paired-samples *t*-tests on participant means. Effect sizes were expressed as Hedges’ $g_{z}$, calculated as the mean paired difference divided by the standard deviation of the paired differences and corrected for small-sample bias [39]. Ninety-five percent confidence intervals for the population standardized paired difference ($\delta_{z}$) were calculated by inverting the noncentral *t* distribution [40]. The Hedges correction was applied to the point estima*t*e only. Effect magnitudes were interpreted using the mean differences in the original measurement units, standardized paired differences, and their confidence intervals. Categorical magnitude labels were not assigned because interpretation depends on the research context and the standardizer used [39,41]. Positive mean differences (single minus dual) indicate higher values in the single-task condition. The Holm procedure was applied across the 19 paired-samples comparisons to control the family-wise error rate at α = 0.05 [42]. Both unadjusted and Holm-adjusted p-values were reported, and statistical significance was determined using the adjusted p-values. Confidence intervals were not adjusted for multiple comparisons.

#### Bayesian analysis.

Bayesian paired *t*-tests were conducted using a two-sided alternative hypothesis and a zero-centered Cauchy prior with a scale of 0.707 on the standardized effect size [BayesFactor R package, R version 4.5.3; 43]. *BF*_10_ quantified evidence for a condition difference (*H*_1_) relative to no difference (*H*_0_), while $BF_{01}=1/BF_{10}$ quantified evidence for *H*_0_ relative to *H*_1_ [26]. Evidence in favor of *H*_1_ was classified using an adapted scale as weak ($1<BF_{10}\leq 3$), moderate ($3<BF_{10}\leq 10$), strong ($10<BF_{10}\leq 30$), or very strong (*BF*_10_ > 30) [44,45]. Evidence in favor of *H*_0_ was classified as weak (0.33 < *BF*_10_ < 1), moderate ($0.10<BF_{10}\leq 0.33$), strong ($0.03<BF_{10}\leq 0.10$), or very strong ($BF_{10}\leq 0.03$). The standardized effect size (δ) was summarized by its posterior median and 95% central credible interval under *H*_1_ [44]. The tabulated posterior summaries were calculated by numerical integration.

#### Statistical parametric mapping.

The spm1d package (v0.4.53, Python), which supports statistical inference across one-dimensional waveforms, was used to compare VGRF between conditions [46]. VGRF waveforms were expressed in BW units and time-normalized separately for the contact phase (t2 to t4) and landing phase (t5 to VTTS). Each trial was interpolated to 101 equally spaced points using piecewise cubic Hermite interpolation (PCHIP; PchipInterpolator in SciPy 1.17.1) before analysis in spm1d. PCHIP was used to preserve local monotonicity and avoid interpolation-induced overshoot between adjacent samples [47]. Landing waveforms included the constant extension described above when VTTS occurred after the recording ended. The three time-normalized trials were averaged within each participant and condition, yielding 35 waveform pairs per phase.

Normality of the paired-difference waveforms was assessed using the D’Agostino–Pearson *K*^2^ test in spm1d. Critical *K*^2^ thresholds for the 101-point waveforms were determined using random field theory at α = 0.05. The maximum *K*^2^ was 13.29 in the contact phase (critical *K*^2^ = 12.92) and 50.88 in the landing phase (critical *K*^2^ = 14.55). These results indicated departures from normality in both phases. Condition differences were assessed using two-sided paired-samples permutation *t*-tests. For each phase, 10,000 permuta*t*ions were generated by exchanging condition labels within participants, assuming symmetry of the paired-difference waveforms under the null hypothesis. The critical threshold was the 95th percentile of the permutation distribution of the maximum absolute *t* statistic. The family-wise error ra*t*e was controlled at α = 0.05 within each phase. Contiguous parts of the *t*-statistic waveform beyond the positive or negative critical *t*hreshold formed suprathreshold clusters. Cluster-level *p*-values were calculated from permutation distributions of the maximum cluster area beyond the threshold [48].

Bayesian posterior probability maps quantify the probability of a hypothesis given the data at each point along a waveform [25]. Maps were computed using Bayesian paired *t*-tests at each normalized time point, with a point null hypothesis of zero difference and the same two-sided alternative and Cauchy prior described above (BayesFactor R package). Assuming equal prior odds, posterior probabilities were calculated as $P(H_{1}|\text{data})=BF_{10}/(1+BF_{10})$. Maps were thresholded at $P(H_{1}|\text{data})\geq 0.95$.

## Results

Tables 1 and 2 present ICC(2,k), CV, and SEM for the 19 force-time metrics under the single-task and dual-task conditions respectively. Under the single-task condition, 17 metrics had ICCs of 0.812–0.930, with CVs ranging from 2.5% to 24.5%. LTTD had an ICC of 0.477 and a CV of 48.6%, while SLC had an ICC of 0.478 and a CV of 3.0%.

**Table 1:** Intraday reliability of drop jump force-time metrics under the single-task condition.

Metric	ICC (95% CI)	CV% (95% CI)	SEM
JH	0.906 (0.800, 0.954)	6.5 (4.7, 8.4)	2.22
GCT	0.874 (0.781, 0.932)	7.0 (4.8, 9.7)	34.2
RSI	0.885 (0.789, 0.940)	11.5 (8.4, 15.0)	0.160
Avg Pow Braking	0.912 (0.844, 0.953)	6.9 (4.5, 9.7)	4.42
Avg Pow Propulsion	0.886 (0.792, 0.940)	6.8 (5.0, 8.7)	3.41
Delta COM	0.905 (0.833, 0.948)	6.8 (4.7, 9.2)	2.06
PF Braking	0.918 (0.857, 0.956)	8.9 (6.7, 11.3)	0.610
PF Propulsion	0.922 (0.864, 0.958)	7.6 (5.6, 9.8)	0.512
LTTD	0.477 (0.107, 0.714)	48.6 (35.4, 62.3)	9.7
Kvert	0.856 (0.750, 0.922)	18.6 (13.4, 24.5)	94.9
BPT	0.859 (0.754, 0.924)	8.4 (5.6, 11.7)	19.8
PPT	0.889 (0.806, 0.940)	6.3 (4.5, 8.2)	15.2
Braking Imp	0.814 (0.673, 0.900)	3.2 (2.2, 4.5)	0.019
Propulsion Imp	0.930 (0.864, 0.964)	2.5 (1.7, 3.4)	0.015
SLC	0.478 (0.085, 0.719)	3.0 (0.9, 7.0)	0.060
PF Landing	0.850 (0.734, 0.920)	10.4 (7.5, 13.6)	0.707
LR Landing	0.812 (0.671, 0.898)	24.5 (18.8, 30.6)	93.8
Landing Imp	0.896 (0.818, 0.944)	2.6 (2.0, 3.3)	0.048
VTTS	0.896 (0.817, 0.944)	3.2 (2.3, 4.1)	0.047

JH = jump height (cm); GCT = ground contact time (ms); RSI = reactive strength index (m/s); Avg Pow Braking = average braking power (W/kg); Avg Pow Propulsion = average propulsion power (W/kg); Delta COM = peak center of mass displacement (cm); PF Braking = peak force, braking phase (BW); PF Propulsion = peak force, propulsion phase (BW); LTTD = landing peak to takeoff peak timing difference (percentage of GCT); Kvert = normalized vertical stiffness (N/m/kg); BPT = braking phase time (ms); PPT = propulsion phase time (ms); Braking Imp = braking impulse (BW·s); Propulsion Imp = propulsion impulse (BW·s); SLC = spring-like correlation, analyzed as its absolute value so that larger values indicate more spring-like behavior; PF Landing = peak force, rebound landing (BW); LR Landing = loading rate, rebound landing (BW/s); Landing Imp = rebound landing impulse (BW·s); VTTS = vertical time to stabilization (s). ICC = intraclass correlation coefficient ICC(2,k), absolute agreement, k = 3 trials; CV = mean participant coefficient of variation from trials 2 and 3, with participant-bootstrap intervals; SEM = standard error of measurement; BW = body weight; 95% CI = 95% confidence interval.

**Table 2:** Intraday reliability of drop jump force-time metrics under the dual-task condition.

Metric	ICC (95% CI)	CV% (95% CI)	SEM
JH	0.917 (0.839, 0.958)	7.1 (4.9, 9.7)	2.16
GCT	0.883 (0.796, 0.937)	8.1 (6.1, 10.3)	38.1
RSI	0.915 (0.837, 0.956)	11.4 (7.8, 15.4)	0.134
Avg Pow Braking	0.912 (0.844, 0.953)	9.2 (7.0, 11.5)	4.05
Avg Pow Propulsion	0.903 (0.817, 0.950)	7.0 (4.8, 9.5)	3.29
Delta COM	0.929 (0.876, 0.962)	7.1 (5.5, 8.7)	1.91
PF Braking	0.895 (0.817, 0.944)	12.0 (9.3, 15.0)	0.670
PF Propulsion	0.913 (0.848, 0.953)	9.7 (7.3, 12.1)	0.569
LTTD	0.618 (0.333, 0.794)	53.4 (39.9, 67.4)	10.6
Kvert	0.875 (0.781, 0.933)	17.3 (13.0, 22.2)	72.2
BPT	0.905 (0.834, 0.949)	9.7 (7.4, 12.1)	18.2
PPT	0.828 (0.701, 0.907)	6.9 (5.1, 8.9)	22.9
Braking Imp	0.884 (0.797, 0.938)	3.4 (2.6, 4.2)	0.016
Propulsion Imp	0.861 (0.756, 0.925)	3.1 (2.3, 3.9)	0.023
SLC	0.441 (0.035, 0.696)	2.1 (1.3, 3.2)	0.065
PF Landing	0.918 (0.855, 0.956)	7.1 (5.3, 9.0)	0.520
LR Landing	0.817 (0.681, 0.901)	19.8 (15.4, 24.3)	76.0
Landing Imp	0.864 (0.762, 0.926)	2.9 (2.2, 3.7)	0.053
VTTS	0.873 (0.777, 0.931)	3.3 (2.5, 4.1)	0.049

JH = jump height (cm); GCT = ground contact time (ms); RSI = reactive strength index (m/s); Avg Pow Braking = average braking power (W/kg); Avg Pow Propulsion = average propulsion power (W/kg); Delta COM = peak center of mass displacement (cm); PF Braking = peak force, braking phase (BW); PF Propulsion = peak force, propulsion phase (BW); LTTD = landing peak to takeoff peak timing difference (percentage of GCT); Kvert = normalized vertical stiffness (N/m/kg); BPT = braking phase time (ms); PPT = propulsion phase time (ms); Braking Imp = braking impulse (BW·s); Propulsion Imp = propulsion impulse (BW·s); SLC = spring-like correlation, analyzed as its absolute value so that larger values indicate more spring-like behavior; PF Landing = peak force, rebound landing (BW); LR Landing = loading rate, rebound landing (BW/s); Landing Imp = rebound landing impulse (BW·s); VTTS = vertical time to stabilization (s). ICC = intraclass correlation coefficient ICC(2,k), absolute agreement, k = 3 trials; CV = mean participant coefficient of variation from trials 2 and 3, with participant-bootstrap intervals; SEM = standard error of measurement; BW = body weight; 95% CI = 95% confidence interval.

Under the dual-task condition, the same 17 metrics had ICCs of 0.817–0.929, with CVs ranging from 2.9% to 19.8% (Table 2). LTTD had an ICC of 0.618 and a CV of 53.4%, while SLC had an ICC of 0.441 and a CV of 2.1%.

Tables 3 and 4 present the descriptive statistics and frequentist and Bayesian comparisons for the 19 force-time metrics. After Holm adjustment, significant condition differences were detected for 10 of the 19 force-time metrics. JH, RSI, and average propulsion power showed the largest condition effects. JH significantly decreased from 22.53 ± 4.77 cm under single-task conditions to 20.23 ± 4.79 cm under dual-task conditions (*p* < 0.001, $g_{z}$ = 1.40, *BF*_10_ = 1.76 × 10^7^). RSI significantly decreased from 0.909 ± 0.293 to 0.781 ± 0.288 m/s (*p* < 0.001, $g_{z}$ = 1.04, *BF*_10_ = 45,943). Average propulsion power significantly decreased from 32.40 ± 6.25 to 29.47 ± 6.64 W/kg (*p* < 0.001, $g_{z}$ = 1.05, *BF*_10_ = 55,419). Each of these three metrics fell under dual-task conditions in 31 of 35 participants and rose in four. Participant-level distributions are shown in Fig 2. Fig 3 presents group mean BCOM velocity and displacement during ground contact.

**Table 3:** Descriptive statistics and paired-samples *t*-test comparisons of drop jump force-time metrics between single- and dual-task conditions.

Metric	Single (M ± SD)	Dual (M ± SD)	Mean diff (95% CI)	Unadjusted *p*	Holm-adjusted *p*	$g_{z}$ (95% CI)
JH	22.53 ± 4.77	20.23 ± 4.79	2.30 (1.75, 2.85)	<0.001	<0.001	1.40 (0.96, 1.91)
GCT	263.2 ± 55.6	280.6 ± 65.3	−17.4 (−27.8, −7.1)	0.002	0.025	−0.57 (−0.93, −0.22)
RSI	0.909 ± 0.293	0.781 ± 0.288	0.128 (0.087, 0.169)	<0.001	<0.001	1.04 (0.64, 1.48)
Avg Pow Braking	35.57 ± 9.00	34.03 ± 8.21	1.54 (0.22, 2.86)	0.023	0.211	0.39 (0.05, 0.74)
Avg Pow Propulsion	32.40 ± 6.25	29.47 ± 6.64	2.93 (1.99, 3.86)	<0.001	<0.001	1.05 (0.65, 1.49)
Delta COM	19.21 ± 3.87	20.18 ± 4.11	−0.97 (−1.56, −0.37)	0.002	0.031	−0.54 (−0.91, −0.20)
PF Braking	4.793 ± 1.240	4.587 ± 1.192	0.205 (0.018, 0.393)	0.033	0.230	0.37 (0.03, 0.72)
PF Propulsion	4.475 ± 1.080	4.206 ± 1.135	0.270 (0.107, 0.433)	0.002	0.027	0.56 (0.21, 0.92)
LTTD	13.4 ± 7.8	16.6 ± 10.0	−3.2 (−6.3, −0.2)	0.039	0.230	−0.36 (−0.70, −0.02)
Kvert	306.8 ± 146.3	282.7 ± 117.1	24.1 (2.0, 46.3)	0.034	0.230	0.37 (0.03, 0.71)
BPT	126.4 ± 30.4	135.3 ± 34.3	−8.9 (−14.5, −3.3)	0.003	0.033	−0.53 (−0.90, −0.19)
PPT	136.8 ± 26.4	145.3 ± 32.3	−8.5 (−14.0, −3.1)	0.003	0.033	−0.53 (−0.89, −0.18)
Braking Imp	0.344 ± 0.024	0.353 ± 0.028	−0.009 (−0.014, −0.004)	0.001	0.019	−0.58 (−0.95, −0.23)
Propulsion Imp	0.352 ± 0.035	0.349 ± 0.035	0.003 (−0.001, 0.007)	0.153	0.460	0.24 (−0.09, 0.58)
SLC	0.959 ± 0.048	0.949 ± 0.050	0.010 (−0.007, 0.027)	0.231	0.462	0.20 (−0.13, 0.54)
PF Landing	5.090 ± 1.106	4.735 ± 1.080	0.354 (0.130, 0.579)	0.003	0.033	0.53 (0.18, 0.90)
LR Landing	232.6 ± 128.0	200.4 ± 103.3	32.3 (4.1, 60.5)	0.026	0.211	0.38 (0.05, 0.73)
Landing Imp	1.347 ± 0.086	1.327 ± 0.083	0.020 (0.001, 0.039)	0.039	0.230	0.35 (0.02, 0.70)
VTTS	1.135 ± 0.083	1.127 ± 0.080	0.008 (−0.010, 0.026)	0.366	0.462	0.15 (−0.18, 0.49)

JH = jump height (cm); GCT = ground contact time (ms); RSI = reactive strength index (m/s); Avg Pow Braking = average braking power (W/kg); Avg Pow Propulsion = average propulsion power (W/kg); Delta COM = peak center of mass displacement (cm); PF Braking = peak force, braking phase (BW); PF Propulsion = peak force, propulsion phase (BW); LTTD = landing peak to takeoff peak timing difference (percentage of GCT); Kvert = normalized vertical stiffness (N/m/kg); BPT = braking phase time (ms); PPT = propulsion phase time (ms); Braking Imp = braking impulse (BW·s); Propulsion Imp = propulsion impulse (BW·s); SLC = spring-like correlation, analyzed as its absolute value so that larger values indicate more spring-like behavior; PF Landing = peak force, rebound landing (BW); LR Landing = loading rate, rebound landing (BW/s); Landing Imp = rebound landing impulse (BW·s); VTTS = vertical time to stabilization (s). M = mean; SD = standard deviation. Paired-samples *t*-test. $g_{z}$ = bias-corrected standardized paired mean difference. The accompanying 95% CI estimates the population standardized paired difference ($\delta_{z}$) by noncentral *t* inversion. The Hedges correction applies to the point estimate only. Holm adjustmen*t* was applied across all 19 comparisons. Statistical significance was determined using the adjusted p-values. The 95% confidence intervals are unadjusted for multiple comparisons. Positive mean difference = single-task > dual-task. BW = body weight.

**Table 4:** Bayesian paired *t*-test comparisons of drop jump force-time metrics between single- and dual-task conditions.

Metric	*BF*_10_	*BF*_01_	δ (95% CrI)	Evidence
JH	$1.76\times 10^{7}$	<0.001	1.38 (0.91, 1.86)	very strong H1
GCT	20.57	0.049	−0.54 (−0.90, −0.19)	strong H1
RSI	45,943	<0.001	1.01 (0.60, 1.44)	very strong H1
Avg Pow Braking	2.09	0.479	0.37 (0.04, 0.71)	weak H1
Avg Pow Propulsion	55,419	<0.001	1.02 (0.61, 1.45)	very strong H1
Delta COM	14.85	0.067	−0.52 (−0.87, −0.17)	strong H1
PF Braking	1.59	0.631	0.35 (0.02, 0.68)	weak H1
PF Propulsion	17.74	0.056	0.53 (0.18, 0.88)	strong H1
LTTD	1.39	0.719	−0.34 (−0.67, −0.01)	weak H1
Kvert	1.55	0.644	0.34 (0.02, 0.68)	weak H1
BPT	13.03	0.077	−0.51 (−0.86, −0.16)	strong H1
PPT	11.75	0.085	−0.50 (−0.85, −0.16)	strong H1
Braking Imp	26.96	0.037	−0.56 (−0.92, −0.21)	strong H1
Propulsion Imp	0.478	2.09	0.23 (−0.09, 0.55)	weak H0
SLC	0.358	2.79	0.19 (−0.13, 0.51)	weak H0
PF Landing	12.55	0.080	0.51 (0.16, 0.86)	strong H1
LR Landing	1.91	0.523	0.36 (0.03, 0.70)	weak H1
Landing Imp	1.38	0.725	0.33 (0.01, 0.67)	weak H1
VTTS	0.268	3.74	0.14 (−0.18, 0.46)	moderate H0

JH = jump height (cm); GCT = ground contact time (ms); RSI = reactive strength index (m/s); Avg Pow Braking = average braking power (W/kg); Avg Pow Propulsion = average propulsion power (W/kg); Delta COM = peak center of mass displacement (cm); PF Braking = peak force, braking phase (BW); PF Propulsion = peak force, propulsion phase (BW); LTTD = landing peak to takeoff peak timing difference (percentage of GCT); Kvert = normalized vertical stiffness (N/m/kg); BPT = braking phase time (ms); PPT = propulsion phase time (ms); Braking Imp = braking impulse (BW·s); Propulsion Imp = propulsion impulse (BW·s); SLC = spring-like correlation, analyzed as its absolute value so that larger values indicate more spring-like behavior; PF Landing = peak force, rebound landing (BW); LR Landing = loading rate, rebound landing (BW/s); Landing Imp = rebound landing impulse (BW·s); VTTS = vertical time to stabilization (s). Bayesian paired *t*-test; Cauchy(0, 0.707) prior. *BF*_10_ = Bayes factor favoring *H*_1_; *BF*_01_ = Bayes factor favoring *H*_0_. δ = posterior median standardized effect size; 95% CrI = 95% credible interval. Evidence classification: *BF*_10_ > 30, very strong *H*_1_; $10<BF_{10}\leq 30$, strong *H*_1_; $3<BF_{10}\leq 10$, moderate *H*_1_; $1<BF_{10}\leq 3$, weak *H*_1_; 0.33 < *BF*_10_ < 1, weak *H*_0_; $0.10<BF_{10}\leq 0.33$, moderate *H*_0_; $0.03<BF_{10}\leq 0.10$, strong *H*_0_; $BF_{10}\leq 0.03$, very strong *H*_0_. Scale adapted from [44,45]. δ and its credible interval are conditional on *H*_1_. BW = body weight.

**Fig 2:**
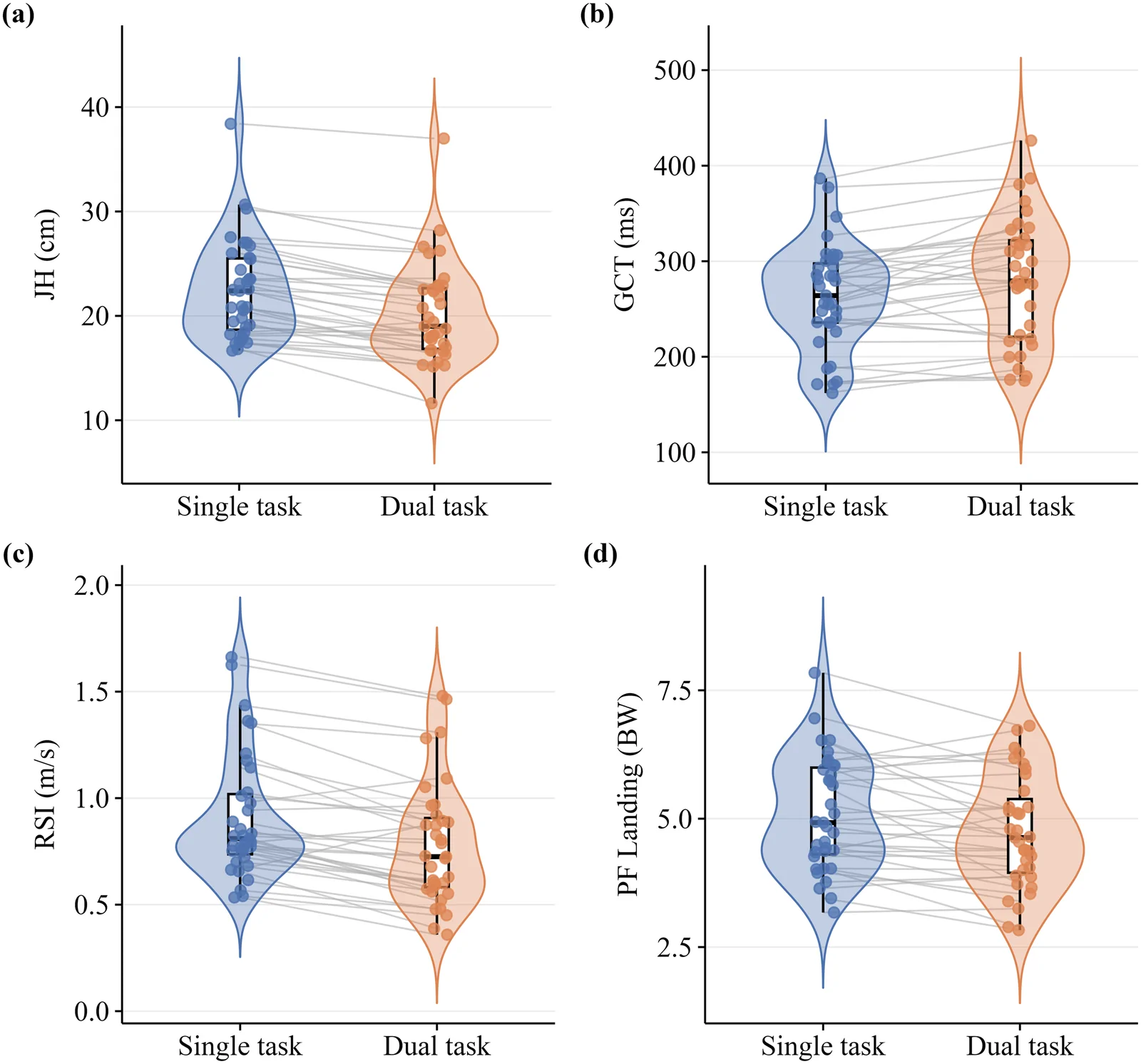
Single-task and dual-task distributions for four force-time metrics selected for graphical presentation. Panels show jump height (JH, a), ground contact time (GCT, b), reactive strength index (RSI, c), and peak landing force (PF Landing, d). BW denotes body weight. Violins show smoothed distributions of participant means (n = 35), and points show individual participant means. Boxes indicate the median and interquartile range, with whiskers extending to the most extreme observations within 1.5 interquartile ranges of the lower and upper quartiles. Gray lines connect observations from the same participant. Blue denotes single-task and orange dual-task.

**Fig 3:**
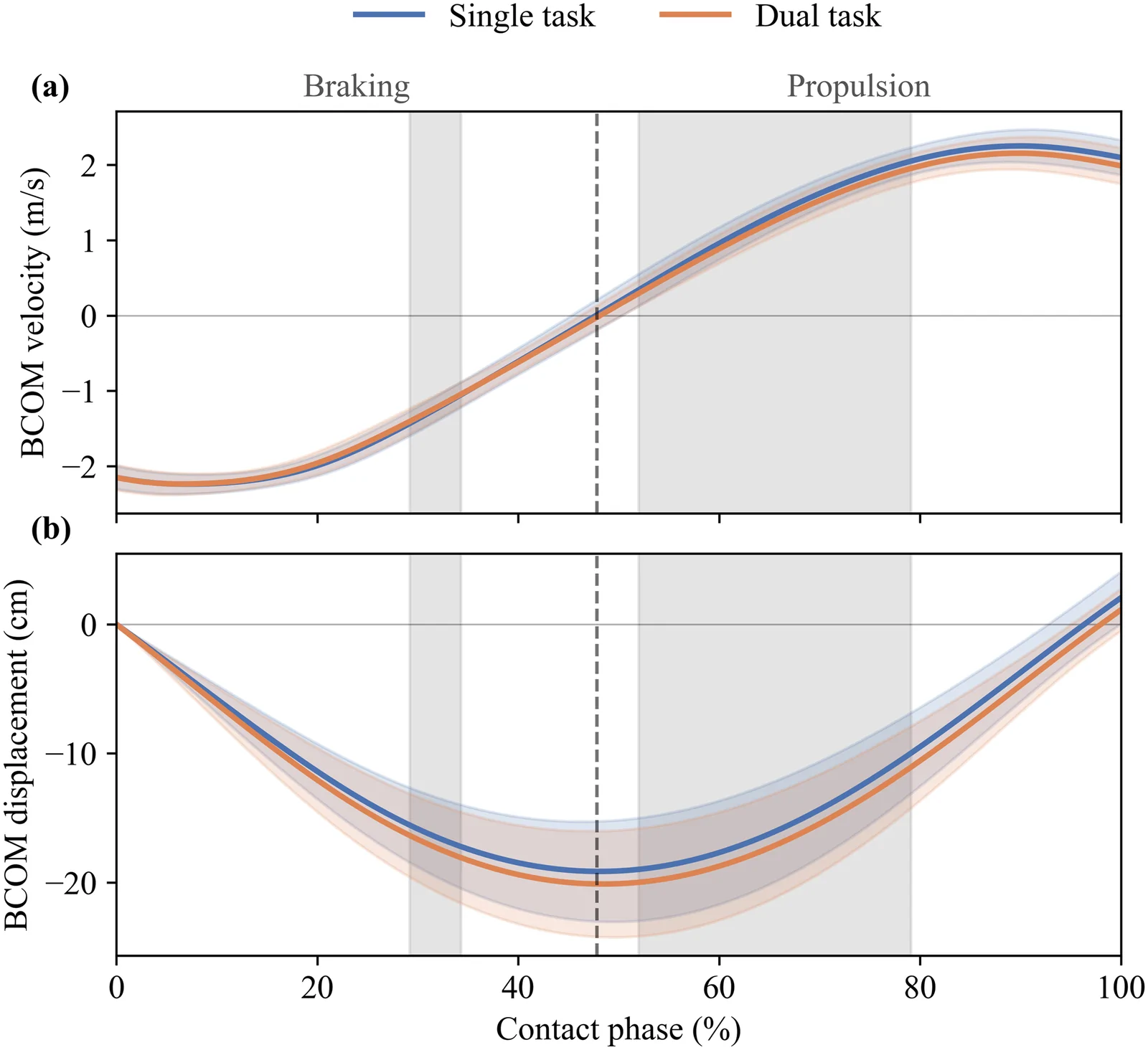
Body center of mass (BCOM) velocity and displacement during ground contact. Mean BCOM velocity (a) and displacement (b), time-normalized from drop landing (0%) to takeoff (100%). Curves and colored bands show group means and ±1 between-participant standard deviation (n = 35). Blue denotes single-task and orange dual-task. Upward velocity and displacement are positive, with displacement set to zero at drop landing. Gray bands show the significant clusters from the vertical ground reaction force analysis in Fig 5b and do not indicate statistical differences in BCOM velocity or displacement. The dashed vertical line marks the mean braking-to-propulsion transition across all trials (47.8% of ground contact).

Average braking power, PF Braking, normalized vertical stiffness, and LTTD did not differ significantly between conditions after Holm adjustment (adjusted p = 0.211, 0.230, 0.230, and 0.230 respectively). Bayesian evidence for these condition differences was weak.

PF Landing was significantly lower under dual-task conditions (adjusted p = 0.033, $g_{z}$ = 0.53, *BF*_10_ = 12.55). Differences in landing loading rate and landing impulse were not statistically significant after Holm adjustment (adjusted p = 0.211 and 0.230 respectively).

No significant condition difference was detected for propulsion impulse (*p* = 0.460, $g_{z}$ = 0.24, *BF*_01_ = 2.09), SLC (*p* = 0.462, $g_{z}$ = 0.20, *BF*_01_ = 2.79), or VTTS (1.135 ± 0.083 vs. 1.127 ± 0.080 s, *p* = 0.462, $g_{z}$ = 0.15, *BF*_01_ = 3.74). Bayesian evidence favoring *H*_0_ was weak for propulsion impulse and SLC and moderate for VTTS. Additional VTTS analyses using an alternative calculation method and different reference-window durations also showed no significant condition differences in the unadjusted comparisons reported in S2 Appendix.

Spring-like behavior was classified as good in 102 of 105 single-task trials (97.1%), moderate in two (1.9%), and poor in one (1.0%). The corresponding counts for the 105 dual-task trials were 95 (90.5%), six (5.7%), and four (3.8%) respectively.

Fig 4 presents the prior and posterior distributions of the standardized effect size for the four force-time metrics selected for graphical presentation, while Fig 5 presents the SPM analysis of contact-phase VGRF. The critical threshold was $t^{*}$ = 3.18 (α = 0.05, two-tailed). Two significant clusters occurred during braking, at 29.2–34.3% of contact (duration = 5.1%, *p* = 0.018, maximum |*t*| = 3.46), and propulsion, at 52.0–79.1% (duration = 27.1%, *p* = 0.002, maximum |*t*| = 3.95). Single-task VGRF was higher than dual-task VGRF in both clus*t*ers. Bayesian posterior probability maps iden*t*ified 22 of 101 normalized time points (21.8%) with $P(H_{1}|\text{data})\geq 0.95$, covering 31–32% and 59–78% of contact (Fig 5c).

**Fig 4:**
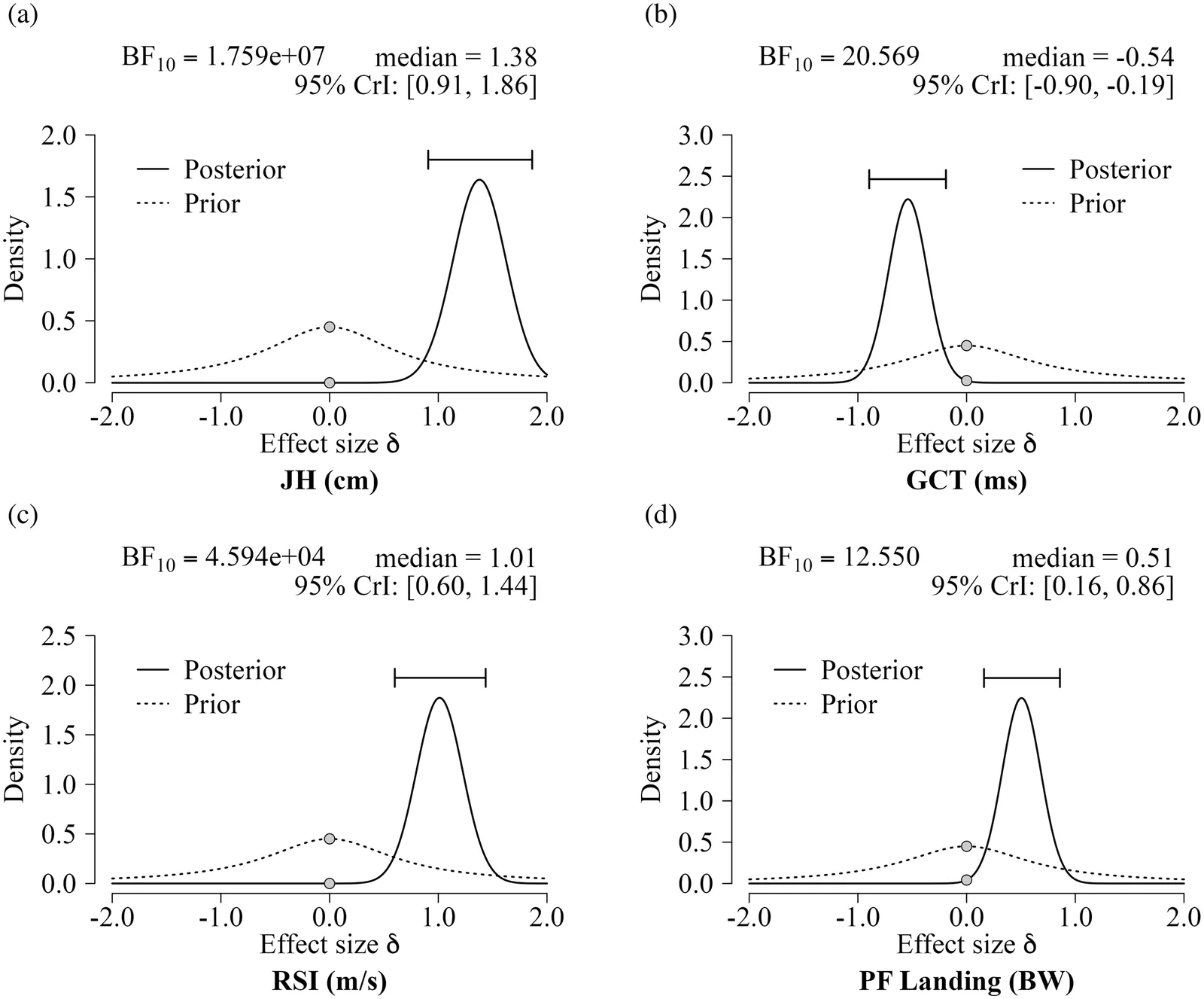
Prior and posterior distributions of standardized paired effect sizes. Panels show jump height (JH, a), ground contact time (GCT, b), reactive strength index (RSI, c), and peak landing force (PF Landing, d). BW denotes body weight. The dotted curve shows the Cauchy prior centered at zero with a scale of 0.707, and the solid curve shows the posterior under the alternative hypothesis (*H*_1_). Positive effect sizes (δ) indicate higher single-task values. Horizontal bars indicate 95% credible intervals (CrIs), and gray points mark the prior and posterior densities at zero. Annotations show the posterior median, CrI, and Bayes factor (*BF*_10_) comparing *H*_1_ (a non-zero difference) with *H*_0_ (no difference). Posterior densities were estimated from 10,000 samples, and the annotated medians and CrIs were calculated by numerical integration, as in Table 4.

**Fig 5:**
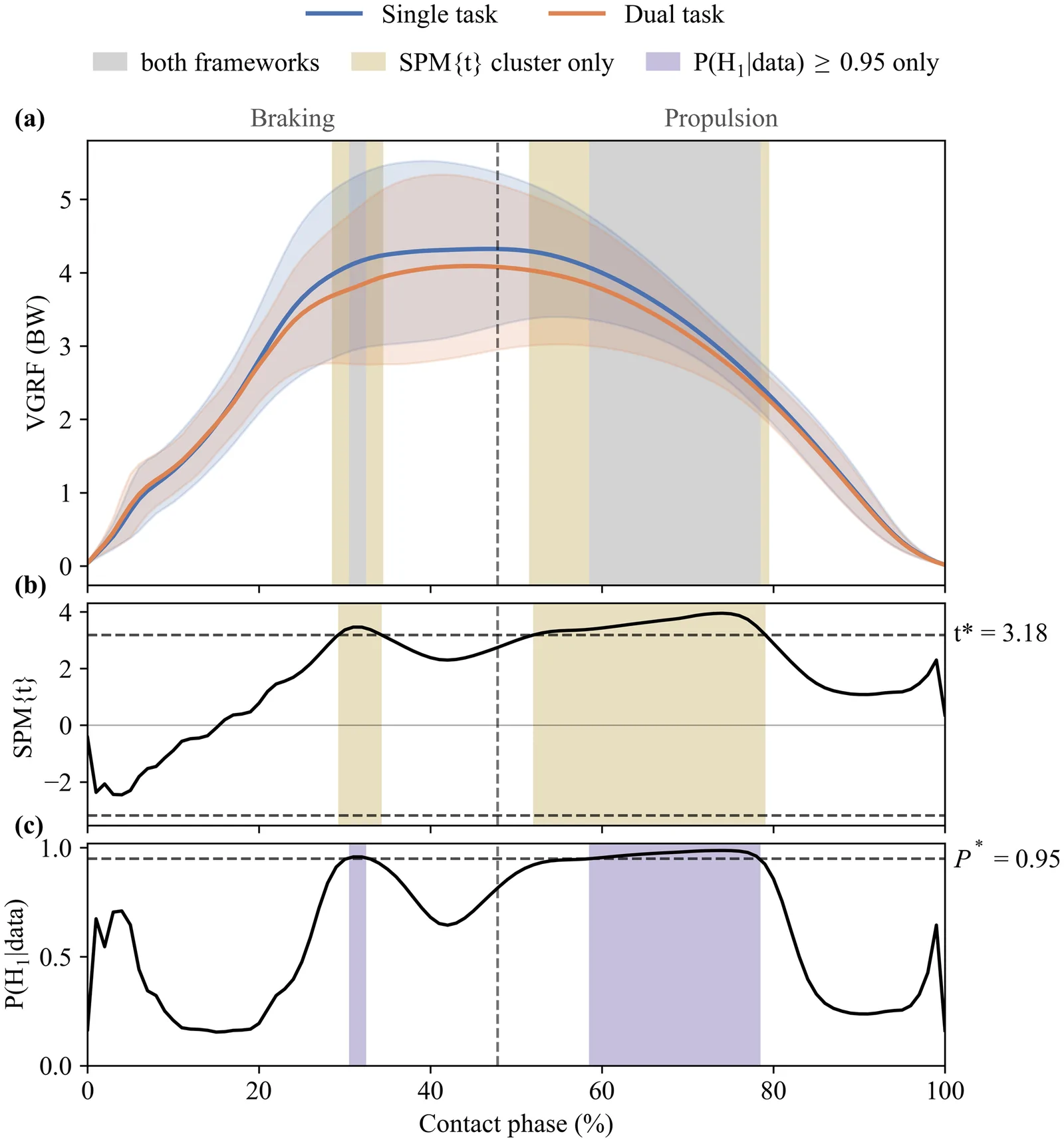
Statistical parametric mapping (SPM) and Bayesian analysis of the contact phase. (a) Mean vertical ground reaction force (VGRF) in body weight (BW) units, time-normalized from drop landing (t2, 0%) to takeoff (t4, 100%). Blue denotes single-task and orange dual-task, with colored bands indicating ±1 standard deviation across 35 participant means. (b) Paired *t*-statistic waveform with dashed permutation thresholds of ±3.18 (α = 0.05, two-sided) and significant clusters shaded gold. Positive *t* values indicate higher single-task VGRF. (c) Posterior probabili*t*y of a non-zero difference between single-task and dual-task conditions, $P(H_{1}|\text{data})$, with the 0.95 criterion shown by a dashed line and qualifying time points shaded purple. In (a), the combined shading is displayed on the 1% time grid, with gray indicating overlap and gold indicating SPM clusters only. All time points meeting the Bayesian criterion fall within significant SPM clusters. The dashed vertical line marks the mean braking-to-propulsion transition across all trials (47.8% of ground contact).

Fig 6 presents the SPM analysis of landing-phase VGRF. The critical threshold was $t^{*}$ = 3.32 (α = 0.05, two-tailed). The first significant cluster occurred during the initial force rise, at 1.3–6.3% of landing (duration = 5.0%, *p* < 0.001, maximum |*t*| = 5.33). Single-task VGRF exceeded dual-task VGRF by 0.33 BW on average within this cluster. The second cluster occurred during impact-force a*tt*enuation, at 15.5–19.6% (duration = 4.1%, *p* = 0.007, maximum |*t*| = 3.70). Dual-task VGRF exceeded single-task VGRF by 0.14 BW on average within this cluster. Bayesian posterior probability maps identified nine of 101 normalized *t*ime points (8.9%) with $P(H_{1}|\text{data})\geq 0.95$, covering 2–6% and 16–19% of landing (Fig 6c).

**Fig 6:**
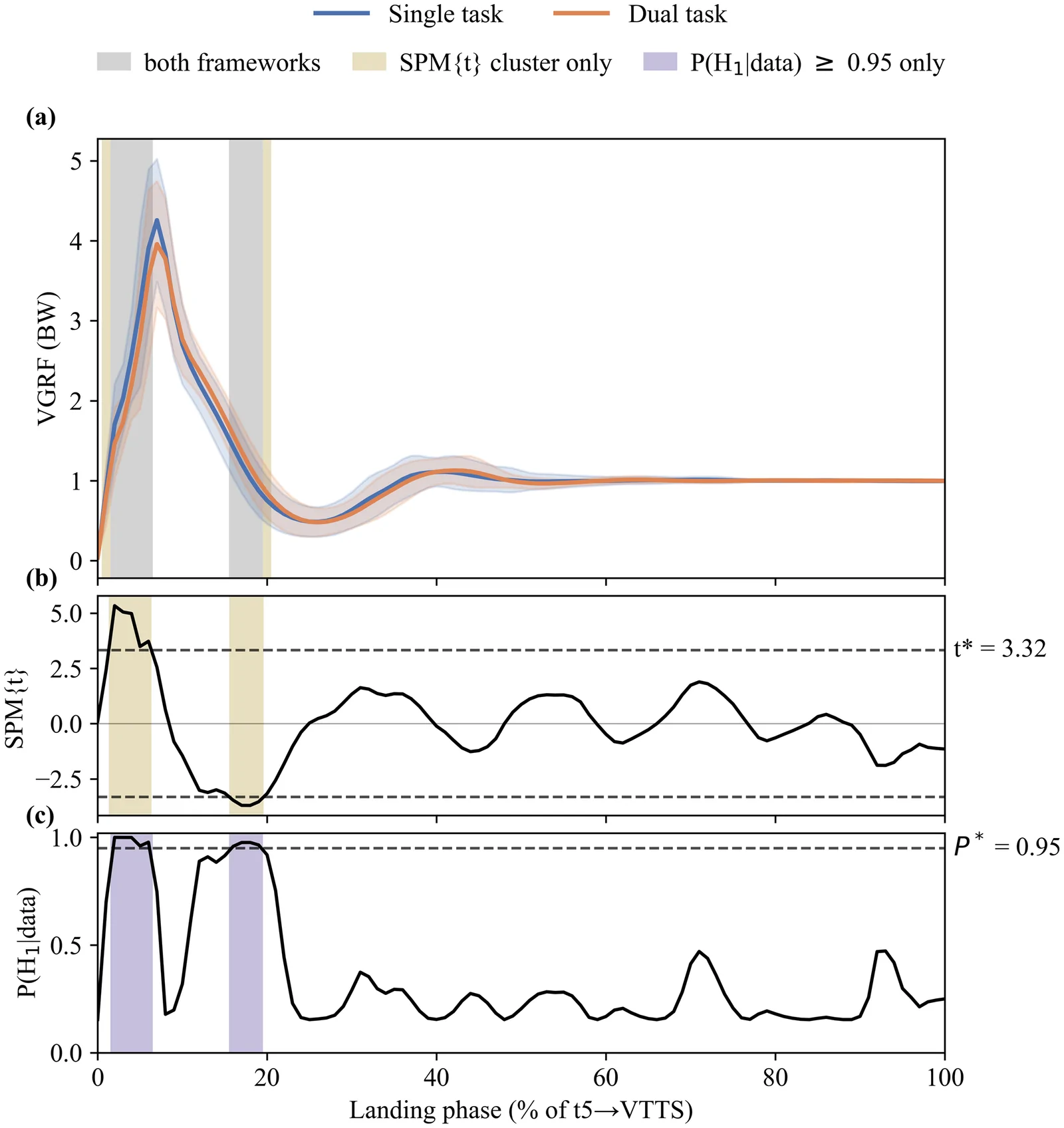
Statistical parametric mapping (SPM) and Bayesian analysis of the landing phase. (a) Mean vertical ground reaction force (VGRF) in body weight (BW) units, time-normalized from rebound landing (t5, 0%) to vertical time to stabilization (VTTS, 100%). Blue denotes single-task and orange dual-task, with colored bands indicating ±1 standard deviation across 35 participant means. (b) Paired *t*-statistic waveform with dashed permutation thresholds of ±3.32 (α = 0.05, two-sided) and significant clusters shaded gold. Positive *t* values indicate higher single-task VGRF. (c) Posterior probabili*t*y of a non-zero difference between single-task and dual-task conditions, $P(H_{1}|\text{data})$, with the 0.95 criterion shown by a dashed line and qualifying time points shaded purple. In (a), the combined shading is displayed on the 1% time grid, with gray indicating overlap and gold indicating SPM clusters only. All time points meeting the Bayesian criterion fall within significant SPM clusters.

## Discussion

In this study, we examined differences in DJ force-time metrics and VGRF waveforms between single- and dual-task conditions. The concurrent CT reduced JH, RSI, and average propulsion power and increased GCT and downward BCOM displacement. SPM identified significant clusters during braking, propulsion, and the subsequent landing. SLC and VTTS showed no significant condition differences, with weak and moderate Bayesian evidence for *H*_0_ respectively. For three-trial mean scores, within-session ICC estimates ranged from 0.812 to 0.930 across both conditions for 17 of the 19 metrics, with lower estimates for SLC and LTTD.

Statistical significance does not necessarily imply strong Bayesian evidence for a condition difference [26]. Although PF Braking and LTTD had unadjusted p-values below 0.05, their *BF*_10_ values of 1.59 and 1.39 provided only weak evidence under the specified prior. Both comparisons were nonsignificant after Holm adjustment (adjusted p = 0.230). These findings provide limited evidence for condition differences and do not establish the absence of an effect.

Our first hypothesis was supported by changes in several force-time metrics indicating deterioration in DJ performance under the dual-task condition, such as lower JH, RSI, and average propulsion power. JH, RSI, and average propulsion power decreased significantly by 2.30 cm, 0.128 m/s, and 2.93 W/kg respectively. These findings are consistent with lower JH during a concurrent CT in previous studies using drop vertical jumps and jump-landing tasks [8,15]. For instance, Shumski et al. [8] reported a 1.84 cm reduction in healthy, recreationally active young adults performing bilateral drop vertical jumps with a concurrent CT very similar to that used in our study. Dai et al. [15] also reported significant JH reductions of approximately 2–3 cm in healthy young recreational athletes performing a double-leg jump-landing task with similar concurrent CTs. The significant JH reductions observed across these studies suggest that a concurrent CT could interfere with maximal jump performance across related jumping tasks in healthy young recreational athletes. However, findings on the effects of concurrent CTs on JH vary across jumping tasks and cognitive demands. For instance, Vivar et al. [49] reported significantly lower JH during countermovement jumps (CMJs) in healthy adult amateur soccer players who responded to a light signal while identifying colors, symbols, and numbers on a screen. In contrast, Memain et al. [50] reported significantly higher JH during single-leg CMJs with a color-identification task in healthy male professional soccer players and those in the final stage of rehabilitation for ACL rupture, knee chondropathy, or muscle injuries. The authors suggested that this visual task may have promoted an external focus of attention and reduced movement inhibition. These contrasting findings may reflect differences in the motor task, cognitive demands, and participant characteristics.

One of the main findings in our study was that lower JH was accompanied by significant increases in GCT, BPT, PPT, and downward BCOM displacement. GCT increased by 17.4 ms. As RSI is the ratio of JH to GCT, the lower RSI reflected both reduced JH and increased GCT. Shumski et al. [8] reported no significant differences in GCT or RSI between single- and dual-task drop vertical jumps. However, Holmes et al. [16] reported significantly longer GCT during DJs when visual or auditory cues indicated whether to jump or land as participants left the box. Braking impulse increased significantly after Holm adjustment, although PF Braking did not differ significantly between conditions. The longer BPT could account for the increase in braking impulse because impulse is the area under the force-time curve. This relationship between force magnitude, application time, and impulse was also recently illustrated by Mavili et al. [51], who reported higher DJ peak forces with shorter GCT and no significant braking or propulsion impulse differences in faster basketball players.

PF Braking was lower under dual-task conditions, although the difference was not statistically significant after Holm adjustment (adjusted p = 0.230). This finding contrasts with the significantly higher peak VGRF reported during the initial landing of a jump-landing task [15] and a DJ [18] under concurrent CTs and during forward drop landings with simulated shooting [33]. Bayesian evidence for the PF Braking difference was weak, and variation in movement direction, landing instructions, and CT demands may contribute to these contrasting findings.

At the subsequent landing, PF Landing was lower under dual-task conditions after Holm adjustment (adjusted p = 0.033). Landing loading rate was calculated over the force rise to PF Landing and should therefore be interpreted alongside the peak force. The loading rate was numerically lower under dual-task conditions, although the difference was not statistically significant after Holm adjustment (adjusted p = 0.211). Lower rebound JH may have contributed to the lower PF Landing through reduced downward velocity at touchdown [1]. However, landing forces also depend on muscle activation and joint motion [3], and the present measurements cannot separate these possible contributions. The SPM results showed lower dual-task VGRF during the initial force rise and higher VGRF during impact-force attenuation, indicating that the condition differences varied across the landing phase. These findings do not establish improved landing control or reduced injury risk.

No significant condition differences were detected for SLC or VTTS. For SLC, within-session ICCs were 0.478 and 0.441 in the single- and dual-task conditions respectively. The limited between-participant variation in SLC may have contributed to these estimates because the ICC depends on between-participant variability relative to measurement error [20]. Bayesian evidence for *H*_0_ was weak (*BF*_01_ = 2.79), and the nonsignificant SLC result does not establish that spring-like behavior was unaffected. VTTS had higher within-session ICCs (0.896 and 0.873 respectively), with moderate Bayesian evidence for *H*_0_ (*BF*_01_ = 3.74) under the specified prior. Good spring-like behavior was observed in 102 single-task trials (97.1%) and 95 dual-task trials (90.5%), indicating a predominantly spring-like force–displacement relationship despite lower jump performance [4]. He et al. [52] also reported no significant difference in time to stabilization during an auditory working-memory task in participants with chronic ankle instability, although their single-leg landing measure required stabilization of all three ground reaction force components. The findings of our study suggest that changes in jump performance could occur without significant changes in these metrics, yet they do not demonstrate that SSC mechanisms or postural control were unaffected under our experimental conditions.

The reductions in key DJ force-time metrics (e.g., JH and RSI) during a concurrent CT may reflect interference with motor preparation. Well-timed muscle activation before touchdown and a rapid transition from braking to propulsion contribute to effective SSC function [2,3]. Maslovat et al. [14] reported reduced motor preparation during a concurrent CT in a wrist-extension reaction-time task. Although that task differs from the DJ, it provides a possible explanation for how a concurrent CT could affect the preparation of a motor response. Altered preparatory muscle activation is one possible explanation for the observed force-time differences, although it was not measured in this study. These differences therefore do not establish whether anticipatory, reflex-mediated, or voluntary control changed under dual-task conditions.

Our expectation of phase-specific VGRF waveform differences was supported by the significant SPM clusters during braking, propulsion, and the subsequent landing. During braking and propulsion, VGRF was lower under dual-task conditions at 29.2–34.3% and 52.0–79.1% of ground contact respectively. The lower VGRF within the propulsion cluster was consistent with the significant reductions in PF Propulsion and average propulsion power. The Bayesian posterior probability maps identified narrower intervals within the significant SPM clusters during braking, propulsion, and the subsequent landing, providing complementary information about the evidence for a condition difference [25]. These findings indicate that the motor performance deterioration was accompanied by phase-specific changes in force application.

Regarding our second hypothesis, 17 of the 19 force-time metrics had within-session ICC estimates of 0.812–0.930 across the single- and dual-task conditions. The ICCs for SLC were 0.478 and 0.441, and those for LTTD were 0.477 and 0.618 in the single- and dual-task conditions respectively. These estimates describe the reliability of three-trial mean scores and should be interpreted alongside their 95% confidence intervals, SEM, and CV, with consideration of the intended use of the measurements [20]. Under conventional single-task testing, Celik et al. [21] reported intraday absolute-agreement ICCs of 0.78–0.89 across 16 DJ force-time metrics. For VTTS, their intraday ICC of 0.89 was similar to the estimates in the present study. In contrast, Flanagan et al. [30] reported lower VTTS reliability during DJs. Differences in arm constraints and stabilization algorithms may contribute to this variation, emphasizing the need to interpret reliability in relation to the testing and analysis procedures.

SLC had CVs of 3.0% and 2.1% in the single- and dual-task conditions respectively. The corresponding LTTD CVs were 48.6% and 53.4%, indicating substantial within-participant variability relative to the mean. Relative reliability and absolute measurement error should be considered together because the ICC depends on between-participant variability [20]. For RSI, ICCs of 0.885 and 0.915 were accompanied by CVs of 11.5% and 11.4% in the single- and dual-task conditions respectively. The ICCs describe three-trial mean scores, while the reported CVs are averages of participant CVs calculated from the second and third trials [37]. These averages do not establish the measurement error for a particular athlete [20]. These ICCs therefore do not establish that small changes in an individual athlete can be distinguished from within-session measurement variability. The LTTD difference was not statistically significant after Holm adjustment (adjusted p = 0.230), and Bayesian evidence for a condition difference was weak.

The lower JH and RSI have practical implications for standardizing cognitive demands during DJ testing. The mean reductions in JH (2.30 cm) and RSI (0.128 m/s) were below the corresponding 95% smallest detectable changes for a participant (3.55 cm and 0.26 m/s respectively). These thresholds were estimated as $1.96\times \sqrt{2}\times \text{SEM}/\sqrt{3}$ using the single-task residual SEM and assume independent errors of comparable magnitude [20]. The absolute JH difference exceeded 3.55 cm in nine of 35 participants, and the absolute RSI difference exceeded 0.26 m/s in five of 35 participants. JH and RSI each decreased in 31 of 35 participants. Therefore, inconsistent cognitive demands could affect group comparisons even when the mean decrement is below an individual-change threshold. These findings support standardizing cognitive demands during DJ testing in healthy young recreational athletes. However, statistically significant group differences do not establish that changes of similar magnitude can be detected reliably in an individual athlete. The estimated thresholds describe within-session residual error and should not be interpreted as validated thresholds for longitudinal monitoring.

### Limitations

Our study has several limitations. Participants were healthy young recreational athletes enrolled in coaching education, which limits generalization to other training levels and clinical populations, including individuals following ACL reconstruction. Rehabilitation outcomes, subsequent injuries, and readiness to return to sport were not assessed. The findings support standardizing cognitive demands during performance testing, while the clinical value of this protocol for rehabilitation and return-to-sport decisions remains to be established. In addition, the CT and bilateral DJ performed in the akimbo position represent only one combination of cognitive and motor demands. The arithmetic CT does not fully reproduce the visual information processing, rapid decision-making, and responses to unpredictable events required during sport [8]. Future studies could examine athletes from different sports and competition levels, include clinical populations, and compare CTs that reflect the perceptual and decision-making demands of those sports.

Electromyography (EMG) and joint kinematics were not recorded. Therefore, the observed force-time differences cannot be attributed to specific changes in muscle activation or neural control. Future studies combining force measurements, EMG, and joint kinematics could examine the contribution of preparatory muscle activity to DJ performance under cognitive load.

Reliability was assessed within a single session, with three trials per condition. Between-session reliability and the ability to detect longitudinal changes in individual athletes remain to be established for this dual-task protocol.

CT difficulty was not individually adjusted. Therefore, the same subtraction task may have imposed different cognitive demands across participants. Familiarization and successful task completion do not establish equivalent cognitive demands or indicate how attention was allocated between tasks. The findings therefore describe DJ motor performance under the specified dual-task condition. Future DJ studies could adapt the baseline calibration approach used by Gursoy et al. [13] and assess cognitive accuracy and response rate when the cognitive task is performed alone and concurrently with the DJ. In addition, the analysis was restricted to trials that met the task requirements. Although the overall trial repetition frequency was below 5%, the influence of trial selection on the condition differences was not quantified.

The sample-size calculation assumed a standardized paired effect of $d_{z}$ = 0.50 for a single unadjusted comparison and did not account for multiple comparisons. The calculation therefore did not establish 80% power for the Holm-adjusted analyses at the assumed effect size, and sensitivity to smaller effects may also have been limited [53]. Nonsignificant findings should be interpreted alongside the effect-size confidence intervals and Bayes factors.

A further limitation relates to the sequential estimation method used to estimate VTTS, which was applied over the first 3 s after rebound landing [35]. The available VGRF data and recording duration limited the choice of algorithm, as several alternatives require horizontal forces or reference data from 10–20 s after landing [34]. However, automatic stopping, selected to reflect the practical time constraints of team testing, typically ended recordings 1–2 s after rebound landing. To apply the sequential estimation method, the force signals were extended to 3 s using a constant equal to their final 100 ms mean force. This signal extension has the potential to affect VTTS estimates, landing impulse, and landing waveforms (S2 Appendix).

## Conclusion

In conclusion, our first hypothesis that DJ performance would deteriorate under the dual-task condition was supported by significant differences in several force-time metrics (e.g., JH, RSI, and GCT), with varying effect sizes. Our expectation of phase-specific VGRF waveform differences was also supported by significant SPM clusters during braking, propulsion, and the subsequent landing. For three-trial mean scores, within-session ICC estimates ranged from 0.812 to 0.930 across both conditions for 17 of the 19 metrics, with lower estimates for SLC and LTTD. Bayesian evidence for the condition differences that remained significant after Holm adjustment ranged from strong to very strong under the specified prior. Evidence favoring *H*_0_ was weak for propulsion impulse and SLC and moderate for VTTS. Therefore, combining frequentist, Bayesian, and waveform analyses has the potential to provide a more comprehensive assessment of DJ performance, while these findings support standardizing cognitive demands during testing.

## Supporting information

S1 Appendix Sensitivity of spring-like behavior classifications. (PDF)

S2 Appendix Vertical time to stabilization and sensitivity analyses. (PDF)
