# Supplementary material for: Dual-task interference in the drop jump: Frequentist, Bayesian, and waveform analysis of vertical ground reaction force
Source: PLoS One. 2026 Nov 11;21(11):e0362399. doi: 10.1371/journal.pone.0362399 (PMC13754236; doi:10.1371/journal.pone.0362399)
Supplement: Supplementary file 1 [file pone.0362399.s001.pdf]

S1 Appendix. Sensitivity of spring-like behavior classifications

Spring-like behavior (SLB) describes a force–displacement relationship that approximates that of a linear spring during ground contact. In the drop jump (DJ), SLB classifications combine the spring-like correlation (SLC) between vertical ground reaction force (VGRF) and body center of mass displacement with the presence of an early impact peak (Pedley et al., 2021; Pedley et al., 2022). The purpose of this analysis was to examine how alternative impact-peak definitions affected the SLB classifications in our study.

The published impact-peak definition describes the highest visible transient VGRF peak within the first 20% of ground contact, although a numerical identification rule was not specified (Pedley et al., 2021; Pedley et al., 2022). To operationalize this definition for automated analysis, an impact peak was identified when the global contact-phase VGRF maximum occurred within this interval. The end of the 20% search interval was rounded up to the next sample. Alternative local-peak definitions were examined because a smaller early peak can precede a larger later maximum.

Four alternative rules used peak prominence. Prominence is the difference in VGRF between a local peak and the higher of its two surrounding baseline minima. Prominence was evaluated within the first 60 ms of contact, and the largest value was compared with thresholds of 0.50, 0.20, 0.10, and 0.05 body weights (BW). A value of zero was assigned when no local maximum was detected. The fixed interval was used because ground contact time differed between single- and dual-task conditions. Therefore, the comparison varied both the peak definition and the search interval. All rules used the 100 Hz filtered VGRF signal sampled at 1000 Hz. Trials were classified as good SLB when SLC was below −0.80 and no impact peak was identified.

A local maximum was identified in 120 of the 210 trials within the 60 ms search interval. Prominence was assigned zero in the remaining 90 trials. Impact-peak counts and proportions of good SLB under each definition are presented in Table S1.

Table S1: Impact-peak counts and proportions classified as good SLB using the timing of the global contact-phase maximum (first 20% of contact) or local-peak prominence thresholds of 0.50, 0.20, 0.10, and 0.05 BW (first 60 ms). Each condition included 105 trials.

Criterion	Trials flagged	Good, single-task	Good, dual-task
Contact-phase maximum inside the first 20%	13	97.1%	90.5%
Local peak, prominence $\geq$ 0.50 BW	15	93.3%	90.5%
Local peak, prominence $\geq$ 0.20 BW	29	89.5%	81.0%
Local peak, prominence $\geq$ 0.10 BW	52	79.0%	69.5%
Local peak, prominence $\geq$ 0.05 BW	71	72.4%	58.1%

Impact-peak flags differed among a participant's three trials in 11.4% of the 70 participant–condition combinations under the primary rule. The corresponding percentages were 11.4%, 20.0%, 31.4%, and 37.1% at prominence thresholds of 0.50, 0.20, 0.10, and 0.05 BW respectively. These comparisons describe trial-level classifications. No inferential comparison of the category proportions was performed.

In our study, the higher proportion of good SLB under single-task conditions was consistent across the definitions examined, although the proportions varied with the definition. Therefore, comparisons of SLB classifications across studies should account for the peak definition and search interval.

References

Pedley, J. S., DiCesare, C. A., Lloyd, R. S., Oliver, J. L., Ford, K. R., Hewett, T. E., & Myer, G. D. (2021). Maturity alters drop vertical jump landing force-time profiles but not performance outcomes in adolescent females. *Scandinavian Journal of Medicine & Science in Sports*, *31*(11), 2055–2063. https://doi.org/10.1111/sms.14025

Pedley, J. S., Lloyd, R. S., Read, P. J., Moore, I. S., Myer, G. D., & Oliver, J. L. (2022). A Novel Method to Categorize Stretch-Shortening Cycle Performance Across Maturity in Youth Soccer Players. *Journal of Strength and Conditioning Research*, *36*(9), 2573–2580. https://doi.org/10.1519/JSC.0000000000003900
