# Supplementary material for: Dual-task interference in the drop jump: Frequentist, Bayesian, and waveform analysis of vertical ground reaction force
Source: PLoS One. 2026 Nov 11;21(11):e0362399. doi: 10.1371/journal.pone.0362399 (PMC13754236; doi:10.1371/journal.pone.0362399)
Supplement: Supplementary file 2 [file pone.0362399.s002.pdf]

S2 Appendix. Vertical time to stabilization and sensitivity analyses

Vertical time to stabilization (VTTS) is the time required to regain a stable standing posture after landing, estimated from vertical ground reaction force (VGRF). Fransz et al. (2015) conducted a comprehensive comparison of 18 methods for estimating time to stabilization after single-leg drop-jump landings. Estimates varied across methods, and trial length generally had a greater effect than sampling rate or filter settings. The purpose of this analysis was to examine how the calculation method and analysis interval affected VTTS estimates and the comparison between single- and dual-task conditions in our study.

Four of the methods examined by Fransz et al. (2015) use VGRF alone, which was the only force component available for our analysis. The vertical third-order polynomial method (VTOP1) requires reference intervals at 10–15 and 15–20 s after landing, which were not recorded. For VRAW2, VGRF was required to remain within body weight (BW) ±5% continuously for 1 s. The longest continuous interval within this band in our records was 0.924 s.

Two methods were therefore selected for the VTTS analysis. The sequential estimation method VSA1 was used for the primary analysis, and VRAW1 was evaluated as an alternative. VRAW1 identifies the first sample after the final exceedance of the BW ±5% band, after which VGRF remains within the band until the end of the analyzed interval. All comparisons used the 100 Hz filtered VGRF signal.

To estimate VTTS using sequential estimation, the cumulative mean VGRF was calculated from rebound landing (Colby et al., 1999). The reference mean and standard deviation (SD) were calculated over a 3 s reference window, following the published recording duration and its subsequent application to drop jumps (Celik et al., 2024; Colby et al., 1999). VTTS was the time to the first sample after the cumulative mean last exceeded the reference mean plus 0.25 SD. Identical estimates were obtained in all 210 trials when both thresholds (reference mean ±0.25 SD) were applied.

Automatic stopping was selected to reflect the practical time constraints of team testing, and post-landing recordings lasted 1.02–2.32 s. Each record was therefore extended to 3 s using a constant equal to its final 100 ms mean VGRF. This extension affects the reference mean and SD used for

every estimate and assumes that VGRF would remain at that level. In 21 trials, including 12 single-task and nine dual-task trials, the estimated crossing occurred after the final recorded sample, by an average of 68 ms and a maximum of 218 ms. These crossings depend on the assumed VGRF continuation and are not observed stabilization events. The same extended signal was used for landing impulse and landing waveforms when their endpoints exceeded the recorded interval.

Paired comparisons used each participant's mean across three trials in each condition. VRAW1 was evaluated over the same 3 s analysis interval as the primary method, and sequential estimation was repeated with reference windows of 2.5, 3.5, and 4 s. VTTS estimates and paired comparisons for each method and analysis interval are presented in Table S2.

Table S2: VTTS estimates and paired comparisons under alternative methods and analysis intervals. g_z is Hedges' standardized paired effect size, and BF_{01} is the Bayes factor for no difference relative to a difference between single- and dual-task conditions. The p-values shown here are unadjusted. The primary VTTS comparison was included in the Holm adjustment across the 19 metrics reported in Table 3. The alternative-method and reference-window comparisons were not included in that family.

Method and analysis interval	Single-task mean, s	Dual-task mean, s	Unadjusted p	g_z	BF_{01}
Sequential estimation, 3 s (primary)	1.135	1.127	0.366	0.15	3.74
VRAW1, 3 s	0.690	0.705	0.548	−0.10	4.65
Sequential estimation, 2.5 s	1.001	0.992	0.216	0.21	2.67
Sequential estimation, 3.5 s	1.260	1.250	0.334	0.16	3.54
Sequential estimation, 4 s	1.377	1.367	0.382	0.15	3.84

In the unadjusted comparisons, no significant VTTS difference between single- and dual-task conditions was detected across the examined methods and analysis intervals. However, absolute VTTS estimates depended on these choices and the assumed continuation of the VGRF signal. Therefore, comparisons across studies should account for the calculation method and recording duration.

References

Celik, H., Yildirim, A., Unver, E., Mavili, C., Yilmaz, E., Ozturk, F., Arpinar-Avsar, P., & Alpan Cinemre, S. (2024). Force-Time Analysis of the Drop Jump: Reliability of Jump Measures and Calculation Methods for Measuring Jump Height. *Measurement in Physical Education and Exercise Science*, *28*(2), 119–132. https://doi.org/10.1080/1091367X.2023.2257169

Colby, S. M., Hintermeister, R. A., Torry, M. R., & Steadman, J. R. (1999). Lower limb stability with ACL impairment. *Journal of Orthopaedic & Sports Physical Therapy*, *29*(8), 444–454. https://doi.org/10.2519/jospt.1999.29.8.444

Fransz, D. P., Huurnink, A., De Boode, V. A., Kingma, I., & Van Dieën, J. H. (2015). Time to stabilization in single leg drop jump landings: An examination of calculation methods and assessment of differences in sample rate, filter settings and trial length on outcome values. *Gait & Posture*, *41*(1), 63–69. https://doi.org/10.1016/j.gaitpost.2014.08.018
