# Supplementary material for: Dual-task interference in the drop jump: Frequentist, Bayesian, and waveform analysis of vertical ground reaction force
Source: PLoS One. 2026 Nov 11;21(11):e0362399. doi: 10.1371/journal.pone.0362399 (PMC13754236; doi:10.1371/journal.pone.0362399)
Supplement: Supplementary file 3 [file pone.0362399.s004.pdf]

Dear Dr Hasan Sozen,

Thank you very much for your e-mail dated October 13, 2026, concerning our manuscript (Submission ID: PONE-D-26-46578), together with the comments from the reviewers and the academic editor. It is our pleasure to resubmit the revised version of our manuscript for publication in PLOS ONE.

We also appreciate the detailed comments from the reviewers and the academic editor and have incorporated the suggested changes into the manuscript to the best of our ability. The manuscript has certainly benefited from these suggestions.

The response letter includes our responses to the reviewers' comments. All line numbers refer to the clean revised manuscript.

We appreciate your time and consideration and hope that our responses are satisfactory. Thank you again for thoroughly reviewing our manuscript.

Please let us know if any additional information is required. We look forward to your decision.

Sincerely,

Response to Reviewer #1

We sincerely thank the Reviewer for their time in reviewing our study and providing valuable comments and suggestions regarding our manuscript. We have responded to the specific comments of the Reviewer below, with the Reviewer's comments in italics and our responses indented below each comment:

Comments to the Author

1.There are logical loopholes in the classification framework. eg: Spring-like behavior was classified as good (SLC < −0.80 without an impact peak), moderate (SLC < −0.80 with an impact peak), or poor (SLC ≥ −0.80 with an impact peak).

> We thank the Reviewer for raising this point. We agree that the original description did not address the combination of SLC ≥ −0.80 without an impact peak. In Pedley et al. (2022), this combination occurred in only two participants, who were excluded from further analysis. Pedley et al. (2021) included this combination in the moderate category.
>
> We have revised the definition of the moderate category to include both combinations, following Pedley et al. (2021). Under the impact-peak definition used in the main analysis, no trials exhibited SLC ≥ −0.80 without an impact peak. However, when the alternative impact-peak definitions described in S1 Appendix were applied, two trials fell into this combination. The revised classification accommodates these trials within the moderate category. This clarification does not change the classifications or results of the main analysis. We have addressed this issue by revising the text in the Data processing section, which now reads (Lines 228–232):
>
> "Spring-like behavior was classified as good (SLC < −0.80 without an impact peak), moderate (SLC < −0.80 with an impact peak or SLC ≥ −0.80 without an impact peak), or poor (SLC ≥ −0.80 with an impact peak) [4, 32]. Under the impact-peak definition used in the main analysis, the combination of SLC ≥ −0.80 without an impact peak was not observed in any of the 210 trials."
>
> The full references are provided below:
>
> Pedley, J. S., Lloyd, R. S., Read, P. J., Moore, I. S., Myer, G. D., & Oliver, J. L. (2022). A novel method to categorize stretch-shortening cycle performance across maturity in youth soccer players. *Journal of Strength and Conditioning Research, 36*(9), 2573–2580. https://doi.org/10.1519/JSC.0000000000003900

Pedley, J. S., DiCesare, C. A., Lloyd, R. S., Oliver, J. L., Ford, K. R., Hewett, T. E., & Myer, G. D. (2021). Maturity alters drop vertical jump landing force-time profiles but not performance outcomes in adolescent females. *Scandinavian Journal of Medicine & Science in Sports, 31*(11), 2055–2063. https://doi.org/10.1111/sms.14025

2.The reliability of SLC is extremely low, its null results lack explanatory power. The same evidence for the absence of an effect on SLC and VTTS, but "No significant condition difference was detected for ... SLC (p = 0.231, g_z = 0.20, BF_{01} *= 2.79"*

We thank the Reviewer for raising this point. We agree that the relative reliability estimates for SLC should be considered when interpreting its nonsignificant condition difference. Although the original manuscript distinguished weak evidence for H_0 for SLC from moderate evidence for H_0 for VTTS, the implications of their different reliability estimates required further explanation. We have revised the Discussion to connect the reliability findings with the interpretation of these results and to clarify that the SLC finding does not establish the absence of a condition effect (Keysers et al., 2020). We have also clarified that limited between-participant variation may contribute to the SLC reliability estimates, as the ICC is affected by sample variability (Atkinson & Nevill, 1998). The revised text now reads (Lines 459–465):

"No significant condition differences were detected for SLC or VTTS. For SLC, within-session ICCs were 0.478 and 0.441 in the single- and dual-task conditions respectively. The limited between-participant variation in SLC may have contributed to these estimates because the ICC depends on between-participant variability relative to measurement error [20]. Bayesian evidence for H_0 was weak (BF_{01} = 2.79), and the nonsignificant SLC result does not establish that spring-like behavior was unaffected. VTTS had higher within-session ICCs (0.896 and 0.873 respectively), with moderate Bayesian evidence for H_0 (BF_{01} = 3.74) under the specified prior."

The full references are provided below:

Keysers, C., Gazzola, V., & Wagenmakers, E.-J. (2020). Using Bayes factor hypothesis testing in neuroscience to establish evidence of absence. *Nature Neuroscience, 23*(7), 788–799. https://doi.org/10.1038/s41593-020-0660-4

Atkinson, G., & Nevill, A. M. (1998). Statistical methods for assessing measurement error (reliability) in variables relevant to sports medicine. *Sports Medicine, 26*(4), 217–238. https://doi.org/10.2165/00007256-199826040-00002

3.Discrepancy in observed effects.eg: Based on an expected moderate standardized paired effect size (dz = 0.50)… a minimum sample size of 34 participants was required to achieve α = 0.05 and power = 0.80. We recruited 35 volunteers…

We thank the Reviewer for this comment. The value of d_z = 0.50 was the assumed standardized paired effect size used in the a priori sample-size calculation, consistent with the calculation reported by Dai et al. (2018). As explained by Lakens (2022), power calculated a priori is conditional on the assumed effect size, and the true population effect size remains unknown. Effect sizes estimated from the collected data can differ across force-time metrics and from this planning assumption. However, the calculation did not account for multiple comparisons and did not establish 80% power for the Holm-adjusted analyses at the assumed effect size of d_z = 0.50. Sensitivity to smaller effects may also have been limited. As emphasized by Lakens (2022), sample-size planning should account for adjustments to the significance level when testing multiple hypotheses. We have clarified the planning assumption in the Methods, which now reads (Lines 121–124):

"The calculation assumed a standardized paired effect size of d_z = 0.50, consistent with the sample-size calculation of Dai et al. [15]. A minimum of 34 participants was required for 80% power at α = 0.05 for a single paired comparison."

We also added the following text to the Limitations, which now reads (Lines 547–551):

"The sample-size calculation assumed a standardized paired effect of d_z = 0.50 for a single unadjusted comparison and did not account for multiple comparisons. The calculation therefore did not establish 80% power for the Holm-adjusted analyses at the assumed effect size, and sensitivity to smaller effects may also have been limited [53]. Nonsignificant findings should be interpreted alongside the effect-size confidence intervals and Bayes factors."

The full references are provided below:

Dai, B., Cook, R. F., Meyer, E. A., Sciascia, Y., Hinshaw, T. J., Wang, C., & Zhu, Q. (2018). The effect of a secondary cognitive task on landing mechanics and jump performance. *Sports Biomechanics, 17*(2), 192–205. https://doi.org/10.1080/14763141.2016.1265579

Lakens, D. (2022). Sample size justification. *Collabra: Psychology, 8*(1), 33267. https://doi.org/10.1525/collabra.33267

4.Absence of test results of D'Agostino–Pearson K² or p at the Statistical Parametric Mapping.

We thank the Reviewer for noting this omission. We have added the maximum D'Agostino–Pearson K^2 statistic and the corresponding critical value for each phase. We have also clarified that these thresholds were determined using random field theory. The critical threshold was exceeded in both phases, indicating departures from normality. Condition differences were already assessed using paired permutation tests, with symmetry of the paired-difference waveforms assumed under the null hypothesis (Nichols & Holmes, 2002). We have revised the Statistical parametric mapping subsection, which now reads (Lines 297–300):

"Normality of the paired-difference waveforms was assessed using the D'Agostino–Pearson K^2 test in spm1d. Critical K^2 thresholds for the 101-point waveforms were determined using random field theory at $\alpha = 0.05$. The maximum K^2 was 13.29 in the contact phase (critical $K^2 = 12.92$) and 50.88 in the landing phase (critical $K^2 = 14.55$). These results indicated departures from normality in both phases."

The full reference is provided below:

Nichols, T. E., & Holmes, A. P. (2002). Nonparametric permutation tests for functional neuroimaging: A primer with examples. *Human Brain Mapping, 15*(1), 1–25. https://doi.org/10.1002/hbm.1058

5.The default time normalization in spm1d uses cubic spline interpolation. Piecewise Cubic Hermite Interpolation (PCHIP) is an alternative shape-preserving interpolation method. Given their distinct numerical properties, it need explicitly clarify this distinction.

We thank the Reviewer for highlighting this distinction. Time normalization was performed as a separate preprocessing step using PchipInterpolator in SciPy 1.17.1 before statistical analysis in spm1d. PCHIP preserves local monotonicity and avoids overshoot between adjacent samples (Fritsch & Butland, 1984). Unlike conventional cubic spline interpolation, continuity of the second derivative is not imposed. These properties support its use for preserving the local shape of the force-time waveforms during time normalization. We have clarified the implementation and rationale in the Statistical parametric mapping subsection, which now reads (Lines 289–293):

"VGRF waveforms were expressed in BW units and time-normalized separately for the contact phase (t2 to t4) and landing phase (t5 to VTTS). Each trial was interpolated to 101 equally spaced points using piecewise cubic Hermite

interpolation (PCHIP; PchipInterpolator in SciPy 1.17.1) before analysis in spm1d. PCHIP was used to preserve local monotonicity and avoid interpolation-induced overshoot between adjacent samples [47].”

The full reference is provided below:

Fritsch, F. N., & Butland, J. (1984). A method for constructing local monotone piecewise cubic interpolants. *SIAM Journal on Scientific and Statistical Computing, 5*(2), 300–304. https://doi.org/10.1137/0905021

Response to Reviewer #2

We sincerely thank the Reviewer for their time in reviewing our study and providing valuable comments and suggestions regarding our manuscript. We have responded to the specific comments of the Reviewer below, with the Reviewer's comments in italics and our responses indented below each comment:

Comments to the Author

The submitted manuscript addresses an important issue concerning the effects of cognitive load on drop jump (DJ) biomechanics and the reliability of force–time parameters. The combination of discrete parameter analysis and statistical parametric mapping (SPM) of ground reaction force profiles represents a valuable aspect of the study design. Nevertheless, several methodological issues require clarification to strengthen the credibility of the findings and their practical relevance to physiotherapy and the functional assessment of athletes.

We sincerely thank the Reviewer for these encouraging and helpful comments.

1. Lack of an objective assessment of cognitive task performance

In the dual-task condition, participants performed serial subtraction; however, the authors did not describe how cognitive performance was quantitatively assessed during jump execution. Furthermore, trials in which participants made a subtraction error or discontinued the cognitive task were repeated. This approach may introduce trial selection bias and limit the ability to assess the actual dual-task cost.

The authors should clarify how many trials were repeated and consider reporting the number of correct responses, error rates, or subtraction rate. Without such data, it is difficult to establish whether the observed biomechanical changes resulted from cognitive–motor interference or how participants allocated their attentional resources between the two tasks.

> We thank the Reviewer for bringing this concern to our attention. Participants were familiarized with the single- and dual-task procedures before the recorded trials and were able to perform both conditions correctly. All trial recordings were retained in MARS without overwriting. Review of these records indicated an overall repetition frequency below 5%, including restarts due to technical recording issues. This percentage covers all causes of repetition and is not a cognitive-error rate. Although the overall trial repetition frequency was below 5%, the influence of trial selection on the condition differences was not quantified.

Repeating trials that did not meet the task requirements is consistent with previous dual-task studies involving drop vertical jumps and jump-landing tasks. Dai et al. (2018) collected three successful trials per condition after practice, Shumski et al. (2023) collected four successful trials per condition, and Strong and Markström (2025) repeated trials with incorrect motor responses to obtain three successful trials per visual cue. Dai et al. (2018) also reported the number of attempts required to obtain the successful trials and the number of responses produced during the counting tasks. We have clarified the familiarization procedure and the frequency of repeated trials in the Protocol subsection, which now reads (Lines 133–135):

"After the warm-up, participants were given sufficient time to become familiar with the test equipment and procedures and to practice DJs under single- and dual-task conditions. All participants were able to perform both conditions correctly before the recorded trials."

The trial repetition statement now reads (Lines 159–160):

"The overall trial repetition frequency was below 5%, including restarts due to technical recording issues."

Regarding cognitive task difficulty, the same subtraction rule was used for all participants. Previous dual-task studies involving drop vertical jumps and jump-landing tasks have also used shared cognitive task rules without describing calibration to each participant's baseline cognitive capacity. These included counting backward by ones or sevens (Dai et al., 2018), subtraction by sixes or sevens (Shumski et al., 2023), and visual decision-making and memory tasks (Strong & Markström, 2025). In our previous study, Gursoy et al. (2022), cognitive task difficulty was calibrated using seated one-minute arithmetic trials, and the individualized difficulty levels were then applied during 60-second postural trials. In the present study, the complete DJ task took approximately 10 s. The individualized calibration procedure was not implemented. However, the use of a shared task rule does not ensure equivalent cognitive demands across participants. We agree that successful task completion alone does not fully characterize cognitive–motor interference or the allocation of attention between tasks. We have therefore expanded the Limitations, which now reads (Lines 539–546):

"CT difficulty was not individually adjusted. Therefore, the same subtraction task may have imposed different cognitive demands across participants. Familiarization and successful task completion do not establish equivalent cognitive demands or indicate how attention was allocated between tasks. The findings therefore describe

DJ motor performance under the specified dual-task condition. Future DJ studies could adapt the baseline calibration approach used by Gursoy et al. [13] and assess cognitive accuracy and response rate when the cognitive task is performed alone and concurrently with the DJ. In addition, the analysis was restricted to trials that met the task requirements. Although the overall trial repetition frequency was below 5%, the influence of trial selection on the condition differences was not quantified."

The full references are provided below:

Dai, B., Cook, R. F., Meyer, E. A., Sciascia, Y., Hinshaw, T. J., Wang, C., & Zhu, Q. (2018). The effect of a secondary cognitive task on landing mechanics and jump performance. *Sports Biomechanics, 17*(2), 192–205. https://doi.org/10.1080/14763141.2016.1265579

Shumski, E. J., Lempke, L. B., Johnson, R. S., Oh, J., Schmidt, J. D., & Lynall, R. C. (2023). Jump height and hip power decrease during cognitive loading regardless of sex: Implications for sport performance metrics. *Journal of Strength and Conditioning Research, 37*(4), 793–798. https://doi.org/10.1519/JSC.0000000000004322

Strong, A., & Markström, J. L. (2025). Adding secondary cognitive tasks to drop vertical jumps alters the landing mechanics of athletes with anterior cruciate ligament reconstruction. *Journal of Biomechanics, 180*, 112496. https://doi.org/10.1016/j.jbiomech.2025.112496

Gursoy, Z. G., Yilmaz, U., Celik, H., Arpinar-Avsar, P., & Kirazci, S. (2022). Effect of individualized cognitive and postural task difficulty levels on postural control during dual task condition. *Gait & Posture, 96*, 1–8. https://doi.org/10.1016/j.gaitpost.2022.05.001

2. Insufficient control of trial order and potential learning and fatigue effects

The authors state that the single-task and dual-task conditions were interleaved and that their order varied between participants. However, it remains unclear whether the order of conditions was randomised or counterbalanced.

With only three trials performed under each condition, learning effects, adaptation to the cognitive task, and accumulating fatigue may have influenced the results. The authors should provide a more detailed description of how trial order was determined and how these potential confounding factors were controlled.

We thank the Reviewer for requesting this clarification. The order of the six trials, comprising three single-task and three dual-task trials, was randomized for each participant using the random.org sequence generator. The same generator was used in our previous study (Tasci et al., 2024). Consecutive trials of the same condition were permitted. We agree that the term “interleaved” did not adequately describe this procedure and have replaced it with an explicit description of randomization.

Participants were familiarized with both conditions before testing, and approximately one minute of rest was provided between trials. These procedures were intended to limit potential learning and fatigue effects, although they cannot establish that such effects were absent. We have clarified the Protocol subsection, which now reads (Lines 137–140):

“The order of the six trials was randomized for each participant using the random.org sequence generator (https://www.random.org/sequences/). Consecutive trials of the same condition were permitted. Approximately one minute of rest was provided between trials.”

The full reference is provided below:

Tasci, S., Celik, H., & Kirazci, S. (2024). Effects of environmental and task related conditions on postural control under concurrent visual feedback. *Human Movement Science, 94*, 103186. https://doi.org/10.1016/j.humov.2024.103186

3. Limitations in the interpretation of neuromuscular control mechanisms

The authors associate the analysis of the early ground contact phase with anticipatory muscle activation and feedforward control mechanisms. However, it should be emphasised that measuring vertical ground reaction force alone does not allow direct assessment of muscle activation or differentiation between anticipatory, reflex-mediated, and voluntary control mechanisms.

Without simultaneous electromyographic (EMG) measurements, interpretations concerning specific neuromuscular mechanisms should be presented more cautiously.

We thank the Reviewer for highlighting this distinction. We agree that VGRF measurements alone cannot directly assess muscle activation or distinguish between anticipatory, reflex-mediated, and voluntary control mechanisms. Although preparatory muscle activity and reflex responses contribute to landing control (Santello, 2005), their contributions cannot be determined from the force-time differences observed in our study. We have therefore revised the Introduction and Discussion to distinguish the measured biomechanical outcomes from possible

neuromuscular explanations. Altered motor preparation is retained as a possible explanation that requires further investigation. The revised Introduction passage now reads (Lines 29–32):

“Force during early ground contact is influenced by preparatory muscle activation and the geometrical configuration of the lower limbs at touchdown [3]. However, VGRF alone cannot distinguish the contributions of anticipatory, reflex-mediated, and voluntary control.”

The revised Discussion passage now reads (Lines 478–480):

“Altered preparatory muscle activation is one possible explanation for the observed force-time differences, although it was not measured in this study. These differences therefore do not establish whether anticipatory, reflex-mediated, or voluntary control changed under dual-task conditions.”

We have also added an explicit limitation concerning the absence of EMG and joint kinematic measurements, which now reads (Lines 532–535):

“Electromyography (EMG) and joint kinematics were not recorded. Therefore, the observed force-time differences cannot be attributed to specific changes in muscle activation or neural control. Future studies combining force measurements, EMG, and joint kinematics could examine the contribution of preparatory muscle activity to DJ performance under cognitive load.”

The full reference is provided below:

Santello, M. (2005). Review of motor control mechanisms underlying impact absorption from falls. *Gait & Posture, 21*(1), 85–94. https://doi.org/10.1016/j.gaitpost.2004.01.005

4. Need for clarification regarding the interpretation of measurement reliability

The study evaluates within-session reliability based on three trials under each condition. Although the intraclass correlation coefficient (ICC), standard error of measurement (SEM), and coefficient of variation (CV) provide valuable information regarding measurement reliability, they do not automatically establish the suitability of the test for long-term athlete monitoring.

The authors should clearly distinguish between within-session and between-session reliability and discuss whether the magnitude of measurement error is sufficiently small relative to the observed differences between conditions.

We thank the Reviewer for this clarification. We agree that within-session reliability does not establish the suitability of the test for long-term athlete monitoring. All measurements were obtained during a single session, and the ICCs describe the relative reliability of three-trial mean scores within each condition. Between-session reliability was not assessed.

We also agree that measurement error should be interpreted relative to the changes the test is intended to detect (Atkinson & Nevill, 1998). For instance, the mean reductions in JH (2.30 cm) and RSI (0.128 m/s) were smaller than their estimated 95% individual-change thresholds (3.55 cm and 0.26 m/s, respectively). These thresholds were calculated for three-trial means using the single-task residual SEM and assume independent errors of comparable magnitude. Under these assumptions, the absolute mean condition differences for all 19 force-time metrics were below their corresponding estimated 95% individual-change thresholds. The significant group differences therefore do not establish that changes of similar magnitude can be detected reliably in individual athletes. We have clarified the scope of the reliability assessment and expanded the Discussion and Limitations accordingly.

The Reliability subsection now identifies the assessment as “Within-session (intraday) reliability” (Line 249). The revised Discussion passage now reads (Lines 516–520):

“These findings support standardizing cognitive demands during DJ testing in healthy young recreational athletes. However, statistically significant group differences do not establish that changes of similar magnitude can be detected reliably in an individual athlete. The estimated thresholds describe within-session residual error and should not be interpreted as validated thresholds for longitudinal monitoring.”

The added Limitations passage now reads (Lines 536–538):

“Reliability was assessed within a single session, with three trials per condition. Between-session reliability and the ability to detect longitudinal changes in individual athletes remain to be established for this dual-task protocol.”

The full reference is provided below:

Atkinson, G., & Nevill, A. M. (1998). Statistical methods for assessing measurement error (reliability) in variables relevant to sports medicine. *Sports Medicine, 26*(4), 217–238. https://doi.org/10.2165/00007256-199826040-00002

5. Limited generalisability to clinical practice and sports rehabilitation

The study was conducted in a sample of healthy, young, physically active individuals representing different sporting disciplines. Consequently, potential conclusions regarding the application of dual-task testing in rehabilitation, particularly among patients following anterior cruciate ligament (ACL) reconstruction, should be formulated cautiously.

Furthermore, serial subtraction does not fully reflect the perceptual and decision-making demands encountered during sporting activities. These limitations should be explicitly acknowledged in the Discussion. The authors should also distinguish between the potential application of the test for athlete monitoring and its as yet unestablished value in clinical decision-making.

We thank the Reviewer for highlighting these limitations. We agree that the findings should be interpreted within the characteristics of our participants and experimental tasks. Our study examined healthy young recreational athletes and did not assess rehabilitation outcomes, subsequent injuries, or readiness to return to sport. The findings therefore support standardizing cognitive demands during performance testing, while the clinical value of this protocol for rehabilitation and return-to-sport decisions remains to be established.

We also agree that serial subtraction does not fully represent the perceptual and decision-making demands encountered during sport. Shumski et al. (2023), who used a similar arithmetic task during drop vertical jumps, acknowledged that counting backward likely oversimplifies the cognitive processing required during athletic performance. We have clarified the population to which our practical implications apply and expanded the Limitations, which now reads (Lines 522–531):

"Our study has several limitations. Participants were healthy young recreational athletes enrolled in coaching education, which limits generalization to other training levels and clinical populations, including individuals following ACL reconstruction. Rehabilitation outcomes, subsequent injuries, and readiness to return to sport were not assessed. The findings support standardizing cognitive demands during performance testing, while the clinical value of this protocol for rehabilitation and return-to-sport decisions remains to be established. In addition, the CT and bilateral DJ performed in the akimbo position represent only one combination of cognitive and motor demands. The arithmetic CT does not fully reproduce the visual information processing, rapid decision-making, and responses to unpredictable events required during sport [8]. Future studies could examine athletes from different sports and competition levels, include clinical populations, and compare CTs that reflect the perceptual and decision-making demands of those sports."

The full reference is provided below:

Shumski, E. J., Lempke, L. B., Johnson, R. S., Oh, J., Schmidt, J. D., & Lynall, R. C. (2023). Jump height and hip power decrease during cognitive loading regardless of sex: Implications for sport performance metrics. *Journal of Strength and Conditioning Research, 37*(4), 793–798. https://doi.org/10.1519/JSC.0000000000004322

6. Use of general rather than discipline-specific effect size thresholds

The authors use general effect size classifications, including conventional Cohen's thresholds and Munro's classification, to interpret the magnitude of the observed effects. However, these general classifications are not specifically tailored to musculoskeletal research and physiotherapy and may therefore lead to inappropriate interpretations of the practical significance of the findings.

The authors are encouraged to adopt discipline-specific thresholds. According to the recommendations reported in the publication available at https://doi.org/10.1016/j.apmr.2025.05.013, small, medium, and large effect sizes for individual differences (Pearson's r) should correspond to 0.3, 0.5, and 0.6, respectively. For group differences (Cohen's d or Hedges' g), the corresponding thresholds should be 0.1, 0.4, and 0.8.

For analyses of variance (ANOVA) and other statistical procedures reporting eta-squared (η^2) or partial eta-squared (ηp^2), the authors should consider the thresholds of 0.003, 0.039, and 0.138 for small, medium, and large effects, respectively, as reported in https://doi.org/10.3390/stats9030049.

Applying more appropriate effect size interpretation criteria would improve the methodological rigour of the study and facilitate a more meaningful evaluation of the practical relevance of the observed biomechanical differences.

We thank the Reviewer for raising this concern and recommending discipline-specific effect-size guidance. We agree that general magnitude classifications can obscure the distinction between the size of an observed effect and its practical importance. Zieliński (2025) demonstrates that effect-size distributions vary across research contexts, supporting more contextual interpretation.

We have carefully considered the applicability of the recommended thresholds to our analyses. Our condition comparisons report Hedges' g_z, which standardizes the mean paired difference using the standard deviation of the paired differences. This differs from the between-group standardization underlying the recommended

Hedges' g thresholds. As explained by Lakens (2013), the choice of standardizer affects the interpretation and comparability of standardized effects.

We have therefore removed the generic effect-size classifications from the text and Table 3. Interpretation now emphasizes the numerical effect estimates, confidence intervals, and differences in the original measurement units. Practical implications are also considered alongside measurement error, as discussed in our response to Comment 4.

In addition, we have removed the Munro classifications and interpreted reliability using the numerical ICCs and their confidence intervals alongside SEM and CV. We have also revised the evaluation of our second hypothesis to report the numerical reliability estimates and their limitations, with consideration of the intended use of the measurements (Atkinson & Nevill, 1998). Pearson's r thresholds were not applied to ICCs because these statistics describe different properties. Finally, eta-squared and partial eta-squared are not reported in our condition comparisons. Therefore, their interpretation thresholds are not applicable.

The revised Frequentist analysis passage now reads (Lines 269–272):

"Effect magnitudes were interpreted using the mean differences in the original measurement units, standardized paired differences, and their confidence intervals. Categorical magnitude labels were not assigned because interpretation depends on the research context and the standardizer used [39, 41]."

The revised Reliability passage now reads (Lines 261–263):

"Relative reliability was interpreted using the ICC estimates and their 95% confidence intervals. Absolute measurement error was evaluated using SEM and CV, with consideration of the magnitude of the observed condition differences."

The revised Discussion passage now reads (Lines 489–493):

"Regarding our second hypothesis, 17 of the 19 force-time metrics had within-session ICC estimates of 0.812–0.930 across the single- and dual-task conditions. The ICCs for SLC were 0.478 and 0.441, and those for LTTD were 0.477 and 0.618 in the single- and dual-task conditions respectively. These estimates describe the reliability of three-trial mean scores and should be interpreted alongside their 95% confidence intervals, SEM, and CV, with consideration of the intended use of the measurements [20]."

The revised Conclusions passage now reads (Lines 563–565):

"For three-trial mean scores, within-session ICC estimates ranged from 0.812 to 0.930 across both conditions for 17 of the 19 metrics, with lower estimates for SLC and LTTD."

The full references are provided below:

Zieliński, G. (2025). Effect size guidelines for individual and group differences in physiotherapy. *Archives of Physical Medicine and Rehabilitation, 106*(12), 1844–1849. https://doi.org/10.1016/j.apmr.2025.05.013

Lakens, D. (2013). Calculating and reporting effect sizes to facilitate cumulative science: A practical primer for t-tests and ANOVAs. *Frontiers in Psychology, 4*, 863. https://doi.org/10.3389/fpsyg.2013.00863

Atkinson, G., & Nevill, A. M. (1998). Statistical methods for assessing measurement error (reliability) in variables relevant to sports medicine. *Sports Medicine, 26*(4), 217–238. https://doi.org/10.2165/00007256-199826040-00002

7. Lack of correction for multiple statistical comparisons

The authors performed 19 separate statistical tests comparing biomechanical parameters at a significance level of $p < 0.05$, without applying a correction for multiple comparisons. This increases the risk of false-positive findings, particularly for parameters associated with small effect sizes and p-values close to 0.05.

The authors should consider applying a multiple-comparison correction, such as the Holm procedure, or clearly identifying the primary outcomes and treating the remaining analyses as exploratory.

We thank the Reviewer for highlighting the risk associated with multiple statistical comparisons. We agree and have applied Holm's sequentially rejective procedure to the 19 paired-samples comparisons, treating them as one family of tests and controlling the family-wise error rate at $\alpha = 0.05$ (Holm, 1979).

Following adjustment, 10 of the 19 comparisons remained statistically significant. The reductions in JH, RSI, and average propulsion power remained significant, with adjusted p-values below 0.001. However, the differences in average braking power, PF Braking, normalized vertical stiffness, LTTD, landing loading rate, and landing impulse no longer met the significance threshold.

We have revised Table 3 (page 17) to report both unadjusted and Holm-adjusted p-values and updated the Abstract, Results, Discussion, and Conclusions accordingly. Statistical significance for the 19 scalar comparisons now refers to the

Holm-adjusted p-values. Confidence intervals are retained for estimation and are explicitly identified as unadjusted for multiple comparisons. We have removed the previous Limitations paragraph describing the unadjusted analysis and added a sample-size limitation, as explained in our response to Reviewer #1, Comment 3.

The revised Frequentist analysis passage now reads (Lines 273–276):

"The Holm procedure was applied across the 19 paired-samples comparisons to control the family-wise error rate at $\alpha = 0.05$ [42]. Both unadjusted and Holm-adjusted p-values were reported, and statistical significance was determined using the adjusted p-values. Confidence intervals were not adjusted for multiple comparisons."

The full reference is provided below:

Holm, S. (1979). A simple sequentially rejective multiple test procedure. *Scandinavian Journal of Statistics, 6*(2), 65–70. https://www.jstor.org/stable/4615733

8. Discrepancies between high relative reliability and measurement error

Most parameters demonstrate high ICC values; however, some exhibit considerable within-subject variability. For example, the coefficient of variation for the reactive strength index (RSI) was 11.5% in the single-task condition and 11.4% in the dual-task condition, despite high relative reliability.

The authors should discuss in greater detail the extent to which the observed measurement error limits the interpretation of changes at the individual level. This is particularly important if the test is proposed as a tool for monitoring training adaptations or rehabilitation progress.

We thank the Reviewer for highlighting this distinction. We agree that the ICC alone does not establish whether measurement error is sufficiently small to detect changes in an individual athlete. The ICC depends on between-participant variability, and favorable relative reliability can coexist with appreciable variation in repeated measurements (Atkinson & Nevill, 1998).

For RSI, the single- and dual-task ICCs were 0.885 and 0.915 respectively, with corresponding CVs of 11.5% and 11.4%. The ICCs describe the reliability of three-trial mean scores. The reported CVs are the means of individual participant CVs calculated from the second and third trials (Shechtman, 2013). These averages

should not be interpreted as the expected measurement error for every individual athlete (Atkinson & Nevill, 1998).

As discussed in our response to Comment 4, the mean RSI reduction of 0.128 m/s was smaller than the estimated 95% individual-change threshold of 0.26 m/s. This threshold describes within-session residual error under the stated assumptions and has not been validated for longitudinal monitoring. Between-session reliability and sensitivity to changes associated with training or rehabilitation remain to be established.

We have expanded the Discussion to clarify these implications, which now reads (Lines 501–507):

"Relative reliability and absolute measurement error should be considered together because the ICC depends on between-participant variability [20]. For RSI, ICCs of 0.885 and 0.915 were accompanied by CVs of 11.5% and 11.4% in the single- and dual-task conditions respectively. The ICCs describe three-trial mean scores, while the reported CVs are averages of participant CVs calculated from the second and third trials [37]. These averages do not establish the measurement error for a particular athlete [20]. These ICCs therefore do not establish that small changes in an individual athlete can be distinguished from within-session measurement variability."

The full references are provided below:

Atkinson, G., & Nevill, A. M. (1998). Statistical methods for assessing measurement error (reliability) in variables relevant to sports medicine. *Sports Medicine, 26*(4), 217–238. https://doi.org/10.2165/00007256-199826040-00002

Shechtman, O. (2013). The coefficient of variation as an index of measurement reliability. In S. A. R. Doi & G. M. Williams (Eds.), *Methods of clinical epidemiology* (pp. 39–49). Springer Berlin Heidelberg. https://doi.org/10.1007/978-3-642-37131-8_4

9. Limited interpretation of changes in landing parameters

In the dual-task condition, lower peak landing force (PF Landing: 5.090 vs 4.735 BW) and a reduced landing loading rate (LR Landing: 232.6 vs 200.4 BW/s) were observed. The authors should provide a clearer biomechanical interpretation of these findings.

Lower values do not necessarily indicate improved landing control or a reduced risk of injury, as they may result from an altered movement strategy, reduced jump height, or differences in landing velocity.

The interpretation should therefore account for the interdependence of the analysed biomechanical parameters rather than considering individual changes in isolation.

We thank the Reviewer for this clarification. We agree that the landing findings should be interpreted in relation to the preceding jump and the factors influencing impact absorption. Lower rebound JH may have contributed to the lower PF Landing through reduced downward velocity at the subsequent touchdown. Pedley et al. (2017) describe the relationship between fall height, impact velocity, and loading demands. However, landing forces also depend on muscle activation and joint motion (Santello, 2005), and their contributions cannot be separated from the present force measurements.

Following Holm adjustment, PF Landing remained significantly lower under dual-task conditions (adjusted p = 0.033). Landing loading rate was calculated over the force rise to PF Landing and should therefore be interpreted alongside the peak force. The loading rate was numerically lower under dual-task conditions, although the difference was not statistically significant after Holm adjustment (adjusted p = 0.211). We have revised the interpretation accordingly and clarified that these findings do not establish improved landing control or reduced injury risk.

The revised Discussion passage now reads (Lines 449–458):

"At the subsequent landing, PF Landing was lower under dual-task conditions after Holm adjustment (adjusted p = 0.033). Landing loading rate was calculated over the force rise to PF Landing and should therefore be interpreted alongside the peak force. The loading rate was numerically lower under dual-task conditions, although the difference was not statistically significant after Holm adjustment (adjusted p = 0.211). Lower rebound JH may have contributed to the lower PF Landing through reduced downward velocity at touchdown [1]. However, landing forces also depend on muscle activation and joint motion [3], and the present measurements cannot separate these possible contributions. The SPM results showed lower dual-task VGRF during the initial force rise and higher VGRF during impact-force attenuation, indicating that the condition differences varied across the landing phase. These findings do not establish improved landing control or reduced injury risk."

The full references are provided below:

Pedley, J. S., Lloyd, R. S., Read, P., Moore, I. S., & Oliver, J. L. (2017). Drop jump: A technical model for scientific application. *Strength & Conditioning Journal, 39*(5), 36–44. https://doi.org/10.1519/SSC.0000000000000331

Santello, M. (2005). Review of motor control mechanisms underlying impact absorption from falls. *Gait & Posture, 21*(1), 85–94. https://doi.org/10.1016/j.gaitpost.2004.01.005

10. Caution in interpreting the Bayesian analysis

For some parameters, statistically significant differences were accompanied by only weak Bayesian evidence in favour of the alternative hypothesis. Examples include PF Braking ($p = 0.033$; $BF_{10} = 1.59$) and LTTD ($p = 0.039$; $BF_{10} = 1.39$).

The authors should discuss these discrepancies more explicitly and avoid presenting all statistically significant differences as equally convincing. In particular, the distinction between conventional statistical significance and the strength of Bayesian evidence should be clearly acknowledged.

A more balanced interpretation would strengthen the credibility of the biomechanical conclusions and reduce the risk of overstating findings supported by limited evidence.

We thank the Reviewer for highlighting this distinction. We agree that statistical significance should not be interpreted as equivalent to strong Bayesian evidence. P-values and Bayes factors address different inferential questions, and their results should be interpreted together without treating all statistically significant findings as equally convincing (Keysers et al., 2020).

The original PF Braking and LTTD comparisons had unadjusted p-values below 0.05, accompanied by BF_{10} values of 1.59 and 1.39 respectively. These Bayes factors provided only weak evidence for condition differences under the specified prior. Following the Holm adjustment described in our response to Comment 7, both comparisons were nonsignificant (adjusted $p = 0.230$). These findings provide limited evidence for condition differences and do not establish the absence of an effect.

The six comparisons that were no longer significant after Holm adjustment had BF_{10} values of 1.38–2.09, indicating only weak evidence for condition differences under the specified prior. For the ten comparisons that remained significant after Holm adjustment, BF_{10} values ranged from 11.75 to 1.76×10^7, corresponding to strong to very strong evidence under the interpretation scheme stated in the Methods. We have revised the Discussion and Conclusions to distinguish statistical significance

from the strength of Bayesian evidence and to avoid overstating findings supported by limited evidence.

The added Discussion passage now reads (Lines 409–413):

“Statistical significance does not necessarily imply strong Bayesian evidence for a condition difference [26]. Although PF Braking and LTTD had unadjusted p-values below 0.05, their BF_{10} values of 1.59 and 1.39 provided only weak evidence under the specified prior. Both comparisons were nonsignificant after Holm adjustment (adjusted $p = 0.230$). These findings provide limited evidence for condition differences and do not establish the absence of an effect.”

The revised Conclusions passage now reads (Lines 565–567):

“Bayesian evidence for the condition differences that remained significant after Holm adjustment ranged from strong to very strong under the specified prior. Evidence favoring H_0 was weak for propulsion impulse and SLC and moderate for VTTS.”

The full reference is provided below:

Keysers, C., Gazzola, V., & Wagenmakers, E.-J. (2020). Using Bayes factor hypothesis testing in neuroscience to establish evidence of absence. *Nature Neuroscience, 23*(7), 788–799. https://doi.org/10.1038/s41593-020-0660-4
