# Supplementary material for: Dual-task interference in the drop jump: Frequentist, Bayesian, and waveform analysis of vertical ground reaction force
Source: PLoS One. 2026 Nov 11;21(11):e0362399. doi: 10.1371/journal.pone.0362399 (PMC13754236; doi:10.1371/journal.pone.0362399)
Supplement: Peer review history

# Peer review: Decision Letter 0

**DOI:** 10.1371/journal.pone.0362399.r001

**Authors:** Hasan Sozen

12 Oct 2026

Dear Dr. Celik,

Thank you for submitting your manuscript to PLOS One. After careful consideration, we feel that it has merit but does not fully meet PLOS One’s publication criteria as it currently stands. Therefore, we invite you to submit a revised version of the manuscript that addresses the points raised during the review process.

Please submit your revised manuscript by Dec 11 2026 11:59PM. If you will need more time than this to complete your revisions, please reply to this message or contact the journal office at plosone@plos.org. When you're ready to submit your revision, log on to https://www.editorialmanager.com/pone/ and select the 'Submissions Needing Revision' folder to locate your manuscript file.

- A letter that responds to each point raised by the academic editor and reviewer(s). You should upload this letter as a separate file labeled 'Response to Reviewers'.
- A marked-up copy of your manuscript that highlights changes made to the original version. You should upload this as a separate file labeled 'Revised Manuscript with Track Changes'.
- An unmarked version of your revised paper without tracked changes. You should upload this as a separate file labeled 'Manuscript'.

If you would like to make changes to your financial disclosure, please include your updated statement in your cover letter. Guidelines for resubmitting your figure files are available below the reviewer comments at the end of this letter.

If applicable, we recommend that you deposit your laboratory protocols in protocols.io to enhance the reproducibility of your results. Protocols.io assigns your protocol its own identifier (DOI) so that it can be cited independently in the future. For instructions see: https://journals.plos.org/plosone/s/submission-guidelines#loc-laboratory-protocols. Additionally, PLOS One offers an option for publishing peer-reviewed Lab Protocol articles, which describe protocols hosted on protocols.io. Read more information on sharing protocols at https://plos.org/protocols?utm_medium=editorial-email&utm_source=authorletters&utm_campaign=protocols.

As the corresponding author, your ORCID iD is verified in the submission system and will appear in the published article. PLOS supports the use of ORCID, and we encourage all coauthors to register for an ORCID iD and use it as well. Please encourage your coauthors to verify their ORCID iD within the submission system before final acceptance, as unverified ORCID iDs will not appear in the published article. *Only*  the individual author can complete the verification step; PLOS staff *cannot*  verify ORCID iDs on behalf of authors.

We look forward to receiving your revised manuscript.

Kind regards,

Hasan Sozen

Academic Editor

PLOS One

**Journal Requirements:**

When submitting your revision, we need you to address these additional requirements.

1. Please ensure that your manuscript meets PLOS ONE's style requirements, including those for file naming. The PLOS ONE style templates can be found at

https://journals.plos.org/plosone/s/file?id=wjVg/PLOSOne_formatting_sample_main_body.pdf and

https://journals.plos.org/plosone/s/file?id=ba62/PLOSOne_formatting_sample_title_authors_affiliations.pdf

2. Please note that PLOS One has specific guidelines on code sharing for submissions in which author-generated code underpins the findings in the manuscript. In these cases, all author-generated code must be made available without restrictions upon publication of the work. Please review our guidelines at https://journals.plos.org/plosone/s/materials-and-software-sharing#loc-sharing-code and ensure that your code is shared in a way that follows best practice and facilitates reproducibility and reuse.

3. Please update your submission to use the PLOS LaTeX template. The template and more information on our requirements for LaTeX submissions can be found at http://journals.plos.org/plosone/s/latex.

4. If the reviewer comments include a recommendation to cite specific previously published works, please review and evaluate these publications to determine whether they are relevant and should be cited. There is no requirement to cite these works unless the editor has indicated otherwise.

[Note: HTML markup is below. Please do not edit.]

Reviewers' comments:

Reviewer's Responses to Questions

**Comments to the Author**

1. Is the manuscript technically sound, and do the data support the conclusions?

Reviewer #1: Yes

Reviewer #2: Yes

**********

2. Has the statistical analysis been performed appropriately and rigorously?

Reviewer #1: No

Reviewer #2: No

**********

3. Have the authors made all data underlying the findings in their manuscript fully available?

The PLOS Data policy

Reviewer #1: Yes

Reviewer #2: Yes

**********

4. Is the manuscript presented in an intelligible fashion and written in standard English?

Reviewer #1: Yes

Reviewer #2: Yes

**********

Reviewer #1: 1.There are logical loopholes in the classification framework. eg: Spring-like behavior was classified as good (SLC < −0.80 without an impact peak), moderate (SLC < −0.80 with an impact peak), or poor (SLC ≥ −0.80 with an impact peak).

2.The reliability of SLC is extremely low, its null results lack explanatory power. The same evidence for the absence of an effect on SLC and VTTS, but "No significant condition difference was detected for … SLC (p = 0.231, g_z = 0.20, BF₀₁ = 2.79"

3.Discrepancy in observed effects.eg: Based on an expected moderate standardized paired effect size (dz = 0.50)… a minimum sample size of 34 participants was required to achieve α = 0.05 and power = 0.80. We recruited 35 volunteers…

4.Absence of test results of D'Agostino–Pearson K² or p at the Statistical Parametric Mapping.

5.The default time normalization in spm1d uses cubic spline interpolation. Piecewise Cubic Hermite Interpolation (PCHIP) is an alternative shape-preserving interpolation method. Given their distinct numerical properties, it need explicitly clarify this distinction.

Reviewer #2: The submitted manuscript addresses an important issue concerning the effects of cognitive load on drop jump (DJ) biomechanics and the reliability of force–time parameters. The combination of discrete parameter analysis and statistical parametric mapping (SPM) of ground reaction force profiles represents a valuable aspect of the study design. Nevertheless, several methodological issues require clarification to strengthen the credibility of the findings and their practical relevance to physiotherapy and the functional assessment of athletes.

1. Lack of an objective assessment of cognitive task performance

In the dual-task condition, participants performed serial subtraction; however, the authors did not describe how cognitive performance was quantitatively assessed during jump execution. Furthermore, trials in which participants made a subtraction error or discontinued the cognitive task were repeated. This approach may introduce trial selection bias and limit the ability to assess the actual dual-task cost.

The authors should clarify how many trials were repeated and consider reporting the number of correct responses, error rates, or subtraction rate. Without such data, it is difficult to establish whether the observed biomechanical changes resulted from cognitive–motor interference or how participants allocated their attentional resources between the two tasks.

2. Insufficient control of trial order and potential learning and fatigue effects

The authors state that the single-task and dual-task conditions were interleaved and that their order varied between participants. However, it remains unclear whether the order of conditions was randomised or counterbalanced.

With only three trials performed under each condition, learning effects, adaptation to the cognitive task, and accumulating fatigue may have influenced the results. The authors should provide a more detailed description of how trial order was determined and how these potential confounding factors were controlled.

3. Limitations in the interpretation of neuromuscular control mechanisms

The authors associate the analysis of the early ground contact phase with anticipatory muscle activation and feedforward control mechanisms. However, it should be emphasised that measuring vertical ground reaction force alone does not allow direct assessment of muscle activation or differentiation between anticipatory, reflex-mediated, and voluntary control mechanisms.

Without simultaneous electromyographic (EMG) measurements, interpretations concerning specific neuromuscular mechanisms should be presented more cautiously.

4. Need for clarification regarding the interpretation of measurement reliability

The study evaluates within-session reliability based on three trials under each condition. Although the intraclass correlation coefficient (ICC), standard error of measurement (SEM), and coefficient of variation (CV) provide valuable information regarding measurement reliability, they do not automatically establish the suitability of the test for long-term athlete monitoring.

The authors should clearly distinguish between within-session and between-session reliability and discuss whether the magnitude of measurement error is sufficiently small relative to the observed differences between conditions.

5. Limited generalisability to clinical practice and sports rehabilitation

The study was conducted in a sample of healthy, young, physically active individuals representing different sporting disciplines. Consequently, potential conclusions regarding the application of dual-task testing in rehabilitation, particularly among patients following anterior cruciate ligament (ACL) reconstruction, should be formulated cautiously.

Furthermore, serial subtraction does not fully reflect the perceptual and decision-making demands encountered during sporting activities. These limitations should be explicitly acknowledged in the Discussion. The authors should also distinguish between the potential application of the test for athlete monitoring and its as yet unestablished value in clinical decision-making.

6. Use of general rather than discipline-specific effect size thresholds

The authors use general effect size classifications, including conventional Cohen's thresholds and Munro's classification, to interpret the magnitude of the observed effects. However, these general classifications are not specifically tailored to musculoskeletal research and physiotherapy and may therefore lead to inappropriate interpretations of the practical significance of the findings.

The authors are encouraged to adopt discipline-specific thresholds. According to the recommendations reported in the publication available at https://doi.org/10.1016/j.apmr.2025.05.013, small, medium, and large effect sizes for individual differences (Pearson's r) should correspond to 0.3, 0.5, and 0.6, respectively. For group differences (Cohen's d or Hedges' g), the corresponding thresholds should be 0.1, 0.4, and 0.8.

For analyses of variance (ANOVA) and other statistical procedures reporting eta-squared (η²) or partial eta-squared (ηp²), the authors should consider the thresholds of 0.003, 0.039, and 0.138 for small, medium, and large effects, respectively, as reported in https://doi.org/10.3390/stats9030049.

Applying more appropriate effect size interpretation criteria would improve the methodological rigour of the study and facilitate a more meaningful evaluation of the practical relevance of the observed biomechanical differences.

7. Lack of correction for multiple statistical comparisons

The authors performed 19 separate statistical tests comparing biomechanical parameters at a significance level of p < 0.05, without applying a correction for multiple comparisons. This increases the risk of false-positive findings, particularly for parameters associated with small effect sizes and p-values close to 0.05.

The authors should consider applying a multiple-comparison correction, such as the Holm procedure, or clearly identifying the primary outcomes and treating the remaining analyses as exploratory.

8. Discrepancies between high relative reliability and measurement error

Most parameters demonstrate high ICC values; however, some exhibit considerable within-subject variability. For example, the coefficient of variation for the reactive strength index (RSI) was 11.5% in the single-task condition and 11.4% in the dual-task condition, despite high relative reliability.

The authors should discuss in greater detail the extent to which the observed measurement error limits the interpretation of changes at the individual level. This is particularly important if the test is proposed as a tool for monitoring training adaptations or rehabilitation progress.

9. Limited interpretation of changes in landing parameters

In the dual-task condition, lower peak landing force (PF Landing: 5.090 vs 4.735 BW) and a reduced landing loading rate (LR Landing: 232.6 vs 200.4 BW/s) were observed. The authors should provide a clearer biomechanical interpretation of these findings.

Lower values do not necessarily indicate improved landing control or a reduced risk of injury, as they may result from an altered movement strategy, reduced jump height, or differences in landing velocity.

The interpretation should therefore account for the interdependence of the analysed biomechanical parameters rather than considering individual changes in isolation.

10. Caution in interpreting the Bayesian analysis

For some parameters, statistically significant differences were accompanied by only weak Bayesian evidence in favour of the alternative hypothesis. Examples include PF Braking (p = 0.033; BF₁₀ = 1.59) and LTTD (p = 0.039; BF₁₀ = 1.39).

The authors should discuss these discrepancies more explicitly and avoid presenting all statistically significant differences as equally convincing. In particular, the distinction between conventional statistical significance and the strength of Bayesian evidence should be clearly acknowledged.

A more balanced interpretation would strengthen the credibility of the biomechanical conclusions and reduce the risk of overstating findings supported by limited evidence.

**********

what does this mean?). If published, this will include your full peer review and any attached files.

If you choose “no”, your identity will remain anonymous but your review may still be made public.

**Do you want your identity to be public for this peer review?**  For information about this choice, including consent withdrawal, please see our Privacy Policy

Reviewer #1: No

Reviewer #2: No

**********

[NOTE: If reviewer comments were submitted as an attachment file, they will be attached to this email and accessible via the submission site. Please log into your account, locate the manuscript record, and check for the action link "View Attachments". If this link does not appear, there are no attachment files.]

To ensure your figures meet our technical requirements, please review our figure guidelines: https://journals.plos.org/plosone/s/figures

You may also use PLOS’s free figure tool, NAAS, to help you prepare publication quality figures: https://journals.plos.org/plosone/s/figures#loc-tools-for-figure-preparation.

NAAS will assess whether your figures meet our technical requirements by comparing each figure against our figure specifications.

# Author comment: Author response to Decision Letter 1

**DOI:** 10.1371/journal.pone.0362399.r002

16 Oct 2026

Attached as a file.

Attachment Submitted filename: response_letter.pdf

# Peer review: Decision Letter 1

**DOI:** 10.1371/journal.pone.0362399.r003

**Authors:** Hasan Sozen

25 Oct 2026

Dual-Task Interference in the Drop Jump: Frequentist, Bayesian, and Waveform Analysis of Vertical Ground Reaction Force

PONE-D-26-46578R1

Dear Dr. Celik,

We’re pleased to inform you that your manuscript has been judged scientifically suitable for publication and will be formally accepted for publication once it meets all outstanding technical requirements.

Within one week, you’ll receive an e-mail detailing the required amendments. When these have been addressed, you’ll receive a formal acceptance letter and your manuscript will be scheduled for publication.

An invoice will be generated when your article is formally accepted. Please note, if your institution has a publishing partnership with PLOS and your article meets the relevant criteria, all or part of your publication costs will be covered. Please make sure your user information is up-to-date by logging into Editorial Manager at Editorial Manager® and clicking the ‘Update My Information' link at the top of the page. For questions related to billing, please contact billing support.

If your institution or institutions have a press office, please notify them about your upcoming paper to help maximize its impact. If they’ll be preparing press materials, please inform our press team as soon as possible -- no later than 48 hours after receiving the formal acceptance. Your manuscript will remain under strict press embargo until 2 pm Eastern Time on the date of publication. For more information, please contact onepress@plos.org.

Kind regards,

Hasan Sozen

Academic Editor

PLOS One

Additional Editor Comments (optional):

Reviewers' comments:

Reviewer's Responses to Questions

**Comments to the Author**

Reviewer #2: All comments have been addressed

**********

2. Is the manuscript technically sound, and do the data support the conclusions?

Reviewer #2: Yes

**********

3. Has the statistical analysis been performed appropriately and rigorously?

Reviewer #2: Yes

**********

4. Have the authors made all data underlying the findings in their manuscript fully available?

The PLOS Data policy

Reviewer #2: Yes

**********

5. Is the manuscript presented in an intelligible fashion and written in standard English?

Reviewer #2: Yes

**********

Reviewer #2: The authors have addressed my comments and made revisions to the manuscript. Although I do not agree with or accept all of the changes, I have no further comments, as there are no critical errors in the manuscript.

**********

what does this mean?). If published, this will include your full peer review and any attached files.

If you choose “no”, your identity will remain anonymous but your review may still be made public.

**Do you want your identity to be public for this peer review?**  For information about this choice, including consent withdrawal, please see our Privacy Policy

Reviewer #2: No

**********

# Editor report: Acceptance letter

**DOI:** 10.1371/journal.pone.0362399.r004

**Authors:** Hasan Sozen

PONE-D-26-46578R1

PLOS One

Dear Dr. Celik,

I'm pleased to inform you that your manuscript has been deemed suitable for publication in PLOS One. Congratulations! Your manuscript is now being handed over to our production team.

At this stage, our production department will prepare your paper for publication. This includes ensuring the following:

* All references, tables, and figures are properly cited

* All relevant supporting information is included in the manuscript submission,

* There are no issues that prevent the paper from being properly typeset

You will receive further instructions from the production team, including instructions on how to review your proof when it is ready. Please keep in mind that we are working through a large volume of accepted articles, so please give us a few days to review your paper and let you know the next and final steps.

Lastly, if your institution or institutions have a press office, please let them know about your upcoming paper now to help maximize its impact. If they'll be preparing press materials, please inform our press team within the next 48 hours. Your manuscript will remain under strict press embargo until 2 pm Eastern Time on the date of publication. For more information, please contact onepress@plos.org.

You will receive an invoice from PLOS for your publication fee after your manuscript has reached the completed accept phase. If you receive an email requesting payment before acceptance or for any other service, this may be a phishing scheme. Learn how to identify phishing emails and protect your accounts at https://explore.plos.org/phishing.

If we can help with anything else, please email us at customercare@plos.org.

Thank you for submitting your work to PLOS One and supporting open access.

Kind regards,

PLOS One Editorial Office Staff

on behalf of

Assoc. Prof. Hasan Sozen

Academic Editor

PLOS One
